# Modulation of Surgical Site Infection Risk in Spinal and Thoracic Surgeries Through Operative Parameters: A Narrative Review

**DOI:** 10.3390/jcm14228124

**Published:** 2025-11-17

**Authors:** Joanna Suszczyńska, Michał Grabala, Paweł Grabala

**Affiliations:** 1 2nd Clinical Department of General and Gastroenterogical Surgery, Medical University of Bialystok, ul. M. Skłodowskiej-Curie 24a, 15-276 Bialystok, Poland; joanna.suszczynska@uskwb.pl (J.S.); michal@grabala.pl (M.G.); 2 Department of Neurosurgery, Polish-Mother’s Memorial Hospital Research Institute, Rzgowska 281/289, 93-338 Lodz, Poland

**Keywords:** surgical site infection, spinal surgery, thoracic surgery, negative-pressure wound therapy, antibiotic prophylaxis, risk factors

## Abstract

Background: Surgical site infections (SSIs) following spinal and thoracic procedures are associated with prolonged hospitalization and increased morbidity, with incidence rates of 2–15% in spinal surgery and 3–12% in thoracic procedures. Multiple patient-related and procedure-specific factors contribute to wound complications, including diabetes mellitus, obesity, smoking, extended surgical time, excessive tissue dissection, and hardware implantation. Implementing evidence-based prevention and early intervention strategies is essential in high-risk surgical cohorts. Methods: This narrative review followed searches in PubMed, Scopus, ScienceDirect, Cochrane Library, and Embase for studies published between January 2000 and October 2025. Eligible peer-reviewed articles examined SSI incidence, risk factors, or prevention strategies in adult patients undergoing thoracic or spinal surgery. Data extraction focused on operative parameters, antibiotic prophylaxis regimens, negative-pressure wound therapy (NPWT) use, and patient outcomes. Results: Evidence from found recent studies was synthesized. Key findings demonstrated that operative duration > 4 h increased SSI odds by 41% per additional hour, and blood loss > 500 mL doubled infection risk. Prophylactic NPWT reduced deep SSI rates by 50% in high-risk patients (BMI ≥ 35, diabetes, multilevel instrumentation). Intrawound vancomycin powder reduced deep SSIs by 50–60%, particularly in multilevel fusions. Administering prophylactic antibiotics within 30 min of incision was significantly more effective than at 60 min, with a 23% relative risk reduction. Weight-adjusted antibiotic dosing in obese patients lowered SSI rates from 5.1% to 2.9%. Conclusions: Operative parameters strongly predict SSI risk. An integrated risk- and evidence-based approach to wound management following spinal and thoracic surgeries—combining optimized antibiotic prophylaxis, risk-stratified NPWT application, and operative technique modifications—can significantly reduce SSI incidence. Successful implementation requires institutional commitment, multidisciplinary collaboration, and continuous quality improvement to optimize patient outcomes.

## 1. Introduction

Postoperative wound complications remain a significant source of morbidity after spinal and thoracic surgeries, representing one of the most challenging aspects of perioperative care. Deep surgical site infections (dSSIs) and superficial infections (sSSIs) can result in wound dehiscence, delayed healing, hardware exposure, prolonged antibiotic use, and reoperation [1,2,3,4]. The clinical burden of such complications has increased in parallel with the rise in complex surgical interventions, especially in aging and comorbid populations undergoing sophisticated procedures.

The incidence of SSIs following spinal surgery ranges from 2% to 15%, depending on the complexity of the procedure, patient risk factors, and institutional practices [1,5,6,7]. In thoracic surgery, rates range from 3% to 12%, with higher rates observed in procedures involving extensive tissue dissection or prolonged operative times [2,8,9,10,11,12,13,14]. These infections not only compromise patient outcomes but also present significant clinical challenges requiring prolonged treatment and management.

Multiple patient-related and procedure-specific factors contribute to wound complications. Patient factors such as diabetes mellitus, obesity, smoking, immunosuppression, and malnutrition are well-documented contributors [1,2,8,15]. The pathophysiology involves impaired wound-healing mechanisms, compromised immune function, and altered tissue perfusion. Intraoperative determinants include extended surgical time, excessive tissue dissection, poor hemostasis, and hardware implantation, all of which increase postoperative infection risk [6,16,17,18].

The complexity of spinal and thoracic procedures [5,7,19] has evolved significantly over the past two decades. Multilevel spinal fusions, complex deformity corrections, and extensive thoracic resections are now commonplace, each carrying unique risk profiles for postoperative complications. The introduction of minimally invasive techniques has reduced SSI rates in some cases, but many complex cases still require traditional open approaches with their associated risks [5,7,19].

Current clinical guidelines recommend a multidisciplinary approach to reduce infection risks, incorporating perioperative optimization, strict aseptic protocols, and postoperative surveillance [20,21,22,23,24]. The Centers for Disease Control and Prevention (CDC) and the World Health Organization (WHO) have established comprehensive guidelines for SSI prevention, emphasizing the importance of evidence-based interventions throughout the perioperative continuum.

In recent years, novel interventions such as prophylactic negative-pressure wound therapy (NPWT) and intrawound antibiotics have shown promise in reducing SSI incidence [25,26,27,28]. These strategies represent a paradigm shift from reactive treatment to proactive prevention, particularly in high-risk patient populations. The integration of predictive modeling and risk-stratification tools has further improved the ability to identify patients most likely to benefit from these advanced interventions [26,29,30].

This narrative review consolidates current evidence on modifiable intraoperative factors influencing wound outcomes after spinal and thoracic surgeries. The objective is to synthesize data across surgical, pharmacologic, and technological interventions to support more targeted and effective preventive strategies. By examining the interplay between operative parameters and infection risk, we aim to provide clinicians with evidence-based tools for optimizing patient outcomes.

## 2. Methodology

We conducted a comprehensive literature search across several major databases, including PubMed, Scopus, ScienceDirect, the Cochrane Library, and Embase, covering studies published from 1 January 2000, through 1 October 2025. This extended timeframe was intentionally selected to reflect the evolution of surgical techniques and infection prevention strategies over the past 25 years. Our search strategy incorporated a broad set of keywords, combined using Boolean operators to enhance both the sensitivity and specificity of the search. The primary search terms focused on core concepts such as “spinal surgery,” “thoracic surgery,” “surgical site infection,” “SSI,” “wound dehiscence,” and “postoperative infection.” To identify studies involving specific interventions, we included terms such as “NPWT,” “negative-pressure wound therapy,” “vacuum-assisted closure,” “antibiotic prophylaxis,” “intrawound antibiotics,” and “vancomycin powder.” We also used terms related to risk factors, including “blood loss,” “operative time,” “surgical duration,” “instrumentation,” “multilevel fusion,” “risk factors,” “infection prevention,” and “wound complications.” Finally, outcome-related terms such as “reoperation,” “wound healing,” “hospital stay,” and “patient outcomes” were added to capture studies that addressed clinical endpoints. To ensure that the search was as thorough and accurate as possible, we developed the initial strategy in collaboration with a medical librarian and refined it through iterative testing. In addition to database queries, we manually reviewed the reference lists of all included articles and key review papers to identify any additional relevant studies. Furthermore, we examined recent conference abstracts from major surgical and orthopedic societies in an effort to capture emerging data and unpublished findings that might not yet appear in indexed journals. To craft this review we first laid out a set of inclusion rules aiming to capture only studies that were pertinent, scientifically rigorous and of a high standard. Our search was confined to peer-reviewed research articles published in English. Eligible work had to involve adult patients—18 years of age or older—who had undergone thoracic or spinal surgery. We zeroed in on papers that reported the incidence of site infections examined their risk factors or evaluated strategies intended to prevent them. We incorporated a spectrum of study designs—randomized controlled trials, cohort studies, case–control studies, systematic reviews and meta-analyses. To guarantee that our conclusions were both meaningful and statistically robust we limited the analysis, to studies that enrolled at least 20 patients. A clear consistent definition of SSIs— one that follows the Centers for Disease Control and Prevention (CDC) guidelines or a comparable standard—was essential. All SSI classifications adhered to CDC criteria: superficial incisional SSI—an infection that surfaces within a thirty-day window confined solely to the cutaneous surface and its immediate subcutaneous stratum. Deep incisional SSI: an infection that may emerge within a window of 30 to 90 days after the operation (the exact timing is contingent on the type of implant) penetrating the deep soft-tissue strata—namely the fascia and the muscle. Organ/space SSI: an infection emerging between the 30th and 90th day, after surgery involving the organs or cavities accessed during the operation. We also called for a long follow-up period of at least 30 days for superficial infections and 90 days for deep SSIs to ensure outcomes could be evaluated reliably. Conversely any study that fell short of these criteria was excluded. This meant discarding case reports or case series with less than 20 participants, as well as investigations that examined infections unrelated to surgical procedures. Research involving veterinary subjects was likewise beyond the scope of our review. Moreover, articles lacking concrete outcome data on surgical-site infections or employing vague inconsistent definitions were omitted. Ultimately, to safeguard the integrity of our dataset we eliminated studies that displayed statistical reporting along with duplicate publications and any research that involved overlapping patient cohorts. The rationale for the sample-size threshold is that a minimum of twenty patients was selected based on considerations. Many surgical subspecialties end up seeing a modest number of patients for particular procedures. Surgeon or single-center investigations involving fewer than twenty subjects often uncover distinctive technical nuances. This cutoff walks a line, letting a wide net be cast without surrendering methodological exactitude. A sensitivity analysis was slated to gauge the robustness of the findings. Working independently and blind to each other’s judgments, two reviewers painstakingly sifted through every title. Abstracts were assessed using a uniform screening form. Full-text articles that satisfied the eligibility criteria were then examined with a data-extraction instrument custom-built for this review. Data were extracted in a fashion across a wide spectrum of variables. The study-related information captured comprised the type of study, its publication year, the country of origin, and the nature of the setting, versus community. In parallel, patient demographics were recorded, encompassing age, gender, body-mass index (BMI) pre-existing comorbidities and the American Society of Anesthesiologists (ASA) physical-status classification score. Every surgical nuance was recorded with care: the type and scope of the operation, whether the team opted for an open or minimally invasive approach and the deployment of any instrumentation. In operative metrics—total operative duration, estimated blood loss, the number of spinal levels addressed, and the extent of the thoracic resection—were systematically captured. In documenting the interventions, we recorded the antibiotic prophylaxis regimen used and noted whether NPWT was applied and captured any intrawound antibiotics that were administered. For outcomes we tracked the incidence of SSIs, broke them down into superficial, deep or organ-space categories, recorded the time, to infection onset, and counted the rate of reoperations. We also logged the length and completeness of follow-up.

## 3. Preoperative Antibiotic Prophylaxis in Thoracic and Spinal Surgeries

### 3.1. Rationale and Timing

Antibiotic prophylaxis functions as the cornerstone of SSI prevention, in thoracic and surgeries seeking to establish bactericidal tissue concentrations precisely at the moment of incision. The underlying principle is to trim the inoculum down to a level that the host’s immune system can effectively manage. Guidelines invariably call for intravenous antibiotics to be given no later than an hour before the surgical incision and for drugs like vancomycin or fluoroquinolones—whose pharmacokinetic profiles demand longer infusions—within two hours prior to the cut [31,32,33]. The sheer importance of timing simply cannot be overstated. A thirty-minute slip can markedly shift pharmacokinetics resulting in less-than-ideal tissue saturation precisely when the incision is most exposed [21,27,34,35,36,37,38]. Menendez et al. set out to see whether extending antibiotic prophylaxis could actually lower the rate of SSI in patients undergoing instrumented spinal fusion. Their bivariate analysis revealed a significant relationship between the prophylaxis regimen and SSI occurrence—1.7% for the extended protocol versus 6.2% for the standard one (*p* = 0.001). Moreover, the extended regimen was linked to a reduced proportion of SSIs dropping from 4.1% under standard care, to just 0.8% (*p* = 0.001). Their analysis led them to conclude that extending prophylaxis seems to lower the rate of superficial surgical site infections, in spine surgeries that involve instrumentation [22]. In Pivazyan et al.’s meta-analysis, a total of 2446 patients were examined [39] and the practice of extending antibiotics failed to bring about any reduction, in the frequency of deep, superficial or overall SSIs after posterior spinal surgery employing closed suction drainage. Abola et al. [31] investigated whether extending prophylaxis beyond the preoperative dose—by adding a 24 h postoperative course—alters postoperative infection rates. They evaluated 4454 patients, of whom 2672 (≈60%) received the 24 h postoperative antibiotics while 1782 (≈40%) received none after surgery. After propensity-matched analysis, the infection frequencies were essentially the same: 1.8% in the group with antibiotics versus 1.5% in the group without. The timing hinges on making sure enough drug reaches the tissue and maintains a concentration throughout the operation. Cefazolin—by far the commonly used prophylactic antibiotic—attains its peak tissue levels roughly 30 to 60 min after an IV dose. Still, its half-life and distribution can differ, markedly influenced by patient-specific factors such as function, body habitus and overall hemodynamic status. Recent studies indicate that the conventional “within 60 min” benchmark may fall short of delivering outcomes. Retrospective cohort investigations show that administering prophylaxis within a half-hour dramatically cuts the incidence of surgical-site infections in spinal instrumentation cases with several centers reporting a drop from 4.2%, to 2.8% after tightening the timing protocol [17,22,38,40,41,42,43].

### 3.2. Antibiotic Selection and Adjustments

Choosing the prophylactic antibiotic means weighing the expected bacterial flora, local resistance trends, any patient allergies, and the drug’s pharmacokinetic profile. The growing problem of resistance now demands a more nuanced approach to selecting prophylaxis [44]. Standard regimen, first-generation cephalosporins, cefazolin remains the preferred choice because it delivers strong activity against Staphylococcus aureus and the typical skin flora that cause surgical-site infections [9,18,21,42,44]. In terms of tissue distribution, cefazolin outperforms the alternatives, achieving bone and soft-tissue concentrations that are frequently four- to eight-fold higher than the minimum inhibitory concentration, for susceptible organisms. Allergy considerations in patients with a documented β-lactam allergy, the selection of drug hinges on how severe and what type of reaction occurred. When the allergy manifests as a mild rash without any anaphylaxis, clindamycin is a reliable choice, offering strong Gram-positive activity and good bone penetration. Conversely for truly anaphylactic reactions. vancomycin remains the preferred agent, though its extended infusion time means it should be initiated somewhat earlier. MRSA colonization and high-risk settings, and the creeping presence of methicillin-resistant Staphylococcus aureus (MRSA), add a layer of complexity to choices. Preoperative nasal swabbing for MRSA colonization is being embraced by an increasing number of institutions, and a positive result typically triggers the use of vancomycin [18,42,45]. Testing for Staphylococcus aureus may steer prevention tactics against infections (HAIs). A prospective single-center observational cohort at a tertiary care hospital collected swabs from 1000 adult patients. PCR assays were used to detect both methicillin-sensitive S. Aureus (MRSA/MSSA). Overall S. Aureus was present in 33.7% of the cohort with MSSA accounting for 30.9% and MRSA for 2.8% [46]. After adjusting for age and sex having a catheter and requiring nursing care emerged as the strongest predictors of colonization. Testing approaches for SA detection should zero in on groups that are especially vulnerable—those with a heightened chance of contracting HAIs and the accompanying complications. Choosing vancomycin for prophylaxis ought to be anchored in three things: the MRSA carriage rate, the patient’s individual risk profile, and the facility’s own guidelines. In institutions where MRSA colonization climbs above ten percent or when the patient falls into a high-risk category, such as being immunocompromised having a prior MRSA infection or having endured a prolonged hospital stay—using vancomycin prophylactically can be justified even if screening cultures are negative [45,46,47,48,49]. In Weight-Based Dosing, obesity makes careful weight-adjusted dosing essential. The routine cefazolin regimen of 1–2 g often falls short in patients yielding sub-therapeutic tissue concentrations. Current guidance advises a 2 g dose for individuals over 80 kg and a 3 g dose for those exceeding 120 kg [50,51,52,53]. Peppard et al. showed that in otherwise similar obese surgical patients weighing ≥100 kg, the administration of a preoperative cefazolin two-gram dose is associated with a similar rate of SSI compared with patients who received a three-gram dose [5].

### 3.3. Intraoperative Redosing

Keeping levels within the therapeutic window throughout long surgeries is crucial for effective prophylaxis. When an operation drags on and blood loss tops 1500 mL, intra-operative redosing becomes necessary to preserve those concentrations as both extended duration and heavy bleeding markedly lower drug levels in the bloodstream and tissues [54,55,56,57]. Current guidance typically recommends administering cefazolin every three to four hours during the case though some experts advocate a schedule: every two to three hours when there is significant blood loss or major fluid shifts. This re-dosing approach is especially important in spinal fusions and extensive thoracotomies, where operative times frequently exceed four to six hours [56,57,58,59]. The physiological logic behind giving another dose comes down to how the drug cleared and how it can become diluted. In an adult, cefazolin’s half-life is roughly 1.8 h, but that interval can shrink in critically ill patients or those with a hyperdynamic circulation. On top of that, major blood loss and the large fluid volumes used for resuscitation can push levels below the therapeutic threshold [54,55]. Certain researchers probed the link between how long a surgery lasts, the amount of blood lost, and the chance of a surgical-site infection. They uncovered that operations extending beyond five hours and shedding over 800 mL of blood were associated with a markedly higher infection risk—about a 2.4-fold increase with a 95% confidence interval of 1.6 to 3.7 [26,27,60]. Importantly the researchers showed that delivering intra-operative re-doses could blunt much of this added risk.

### 3.4. Duration of Prophylaxis

A time-tested rule in prophylaxis is to keep the regimen brief—short enough to spare patients from side-effects yet long enough to preserve its therapeutic punch. The bulk of evidence now points strongly to stopping prophylactic antibiotics within 24 h after surgery, even for demanding operations that involve spinal hardware or extensive tissue dissection [7,40,61]. The reasoning behind this short-duration strategy is multi-layered. First, the window in which bacteria can slip in— the operation itself and the immediate recovery hours—is the period of greatest infection risk, so extending antibiotics beyond that offers scant extra protection against surgical site infections. Second, the longer antibiotics linger, the odds that resistant strains will emerge is a worry that has grown acute amid the global rise in antimicrobial resistance. Third, when an antibiotic regimen is prolonged, the odds of encountering adverse reactions rise—spanning Clostridioides difficile infection, kidney toxicity and allergic responses—that can muddle the recovery process. Backing this approach, Pivazyan et al. carried out a meta-analysis that pooled seven studies involving a total of 2446 patients. Their results showed that extending antibiotics for patients with surgical drains did not produce a statistically meaningful drop in SSI rates. In fact, the data hinted at a rise in postoperative complications among those receiving the longer courses [39]. In the vein, a propensity-matched analysis by Abola et al. in 2021 showed that extending prophylaxis beyond 24 h provided no clinical benefit [31].

### 3.5. Local and Adjunctive Antibiotic Measures

Delivering antibiotics directly at the surgery site has really taken off as it floods the local area with a strong dose while keeping systemic exposure—and the associated toxicity—well under control. Topical vancomycin is increasingly popular in orthopedic and thoracic surgical practice as a means of decreasing SSIs, particularly dSSIs due to Gram-positive organisms [62]. Although data on efficacy is becoming increasingly strong, its safety profile is still a concern in vulnerable populations like patients with renal insufficiency or those undergoing broad-field procedures involving high antibiotic loads. This section summarizes the existing literature on local toxicity, systemic absorption, adverse reactions, and practical considerations when dose adjustment is needed.

Current meta-analyses and systematic reviews continue to affirm that vancomycin powder is safe and well-tolerated when applied topically to surgical sites. Most research reports no significant local cytotoxicity or impaired wound healing, especially when used at doses ranging from 1 g to 2 g per procedure [63,64,65,66]. Shu and colleagues set out to gauge whether sprinkling vancomycin powder directly onto the field could curb surgical-site infections in spine operations hoping to furnish a foothold for future clinical guidelines. Their meta-analysis comprised a total of 34,301 patients who received the vancomycin powder while 19,508 formed the control arm. The findings revealed a lower SSI rate in the prophylactic vancomycin group compared with the controls. Subgroup analysis revealed that in operations featuring internal fixation, deformity correction or deep-tissue infection, the cohort receiving prophylactic vancomycin powder suffered a substantially lower rate of SSI than the control cohort. In contrast, for patients undergoing spine surgeries, the two groups showed no significant difference in the incidence of superficial tissue infection. Overall, applying vancomycin powder prophylactically in surgery markedly reduces deep-tissue SSI rates with the effect most pronounced in cases involving internal fixation or deformity correction [67]. In a systematic review and meta-analysis, Bakhsheshian et al. studied the effects of the adjunctive local application of vancomycin powder in spine surgery. Fourteen of the studies, 1 randomized controlled trial and 13 comparative studies were eligible for the meta-analysis. The odds of developing a deep infection with intrawound vancomycin powder were 0.23 times the odds of experiencing an infection without intrawound vancomycin. For combined superficial and deep infections, the odds ratio was 0.43. The pooled clinical data supports the use of vancomycin to prevent SSIs in adult spine surgeries [68]. A 2023 meta-analysis by Li et al. [69] published in the European Spine Journal, assessed 8166 spinal deformity surgeries and found no reported local or systemic adverse events related to vancomycin administration, even in pediatric groups.

Similarly, a 2024 review by Daher et al. [70] concluded that topical vancomycin used in randomized controlled trials for spine surgeries did not significantly alter the rate of complications, supporting its overall safety in diverse patient populations. Despite being applied locally, vancomycin can still be absorbed systemically, especially in procedures involving large, open wounds, high tissue vascularity, or significant trauma. A 2022 systematic review by Luo et al. [24] found that vancomycin serum levels after topical application generally remained below nephrotoxic thresholds. However, sporadic elevations did occur, particularly when dosages exceeded 2 g or when patients had pre-existing kidney dysfunction. In rare cases, patients with chronic renal insufficiency developed postoperative acute kidney injury (AKI), which may have resulted from the cumulative effect of both intravenous and topical vancomycin. To minimize these risks, clinicians should closely monitor serum vancomycin levels in high-risk patients who receive both IV and topical vancomycin. For patients with a glomerular filtration rate (GFR) below 30 mL/min/1.73 m^2^ or those on dialysis, a reduced dose—typically under 1 g—is advisable. Routine use of vancomycin powder should also be avoided in open thoracic or spinal wounds with exposed dura, where drug absorption is more unpredictable [24,66,69,70,71,72,73,74,75,76].

Although adverse events are uncommon, several specific complications have been documented. Contact dermatitis or sterile seroma formation at the wound site has occurred in less than 1% of patients across multiple studies. Delayed wound healing has been more frequently observed in individuals receiving high doses of vancomycin (over 2 g), especially during procedures involving orthopedic implants. Additionally, rare systemic hypersensitivity reactions—such as rash, eosinophilia, or anaphylactoid responses—have been reported and should be considered, particularly in patients with a known allergy or prior exposure to vancomycin [66,72,73,74,75,76,77].

In comparison to spinal surgery, the use of topical vancomycin in thoracic procedures—such as thoracotomy or VATS—is less well studied. Nevertheless, emerging evidence suggests that topical vancomycin can help reduce deep sternal or pleural space infections, especially in high-risk cardiac or esophageal surgeries. Due to the higher vascularity of thoracic tissues, there is an increased risk of systemic absorption, which warrants more cautious dosing. In most cases, limiting the dose to under 1 g per wound space is advisable [66,73,74,75,78,79].

Intrawound Vancomycin Powder (IWVP)—a niche trick, now a near-standard maneuver in spinal surgery, involves dusting the wound with the antibiotic just before the final sutures. A growing body of research backs this habit—most studies noting a 50–60% dip in deep surgical-site infections, particularly when the operation spans multiple vertebral levels. In the review by Gande et al., the control group logged 412 SSIs, a 3.8% incidence, while the vancomycin-powder group reported 197 SSIs or 2.3%. Gram-positive SSIs occurred more often in the control arm whereas Gram-negative and polymicrobial SSIs were markedly more frequent in the vancomycin cohort. The odds of a polymicrobial SSI were almost twice as high—about a 93.5% increase—in the vancomycin group. These findings imply that the broad prophylactic use of intrawound vancomycin may actually raise the incidence of polymicrobial SSIs. Accordingly, the application of vancomycin powder should probably be limited to those procedures and patients who sit at the risk of infection [80]. Luo et al. pooled data from 22 trials involving 11,555 patients. They showed that applying vancomycin topically led to a marked drop in overall surgical-site infections and Gram-positive/MRSA infections even though its effect on superficial infections was comparatively modest [24]. He et al.’s meta-analysis examined whether applying vancomycin powder could curb surgical-site infections in operations. Their pooled data showed a reduction in both SSI rates and deep-infection occurrences when the powder was used. In a subgroup focused on procedures, the protective effect of vancomycin powder remained evident [20]. Khan and colleagues set out to answer a question—does placing vancomycin powder directly into the wound lower the rate of surgical site infections (SSIs) in spine surgery? Their pooled analysis covered 2574 patients in the control arm, among whom 106 developed an SSI (4.1%) and 2518 patients who received the powder with 33 infections (1.3%). This translates to a risk reduction of 2.8% and a relative risk drop of roughly 68%. The meta-analysis therefore indicates that intrawound vancomycin powder acts as a factor against SSIs. The subgroup analysis revealed that when vancomycin powder was applied, patients who had implants faced a lower chance of surgical site infection than those who underwent spinal operations without any instrumentation [81]. In practice clinicians typically aim for a dose between one and two grams, titrating it to the size of the field. Worries about local toxicity, allergic episodes or the emergence of resistance have not been substantiated in real-world use though sustained long-term monitoring is still advisable. Irrigation solutions—washing the wound with an antimicrobial fluid while the patient is still on the table adds another layer of bacterial reduction. In a 2022 review and meta-analysis of spinal-surgery irrigation methods, Torres et al. observed that diluted antiseptic rinses appear to be a promising strategy for preventing surgical-site infections [32]. Diluted povidone-iodine (0.35–0.5%) and low-strength chlorhexidine (0.05%) have been shown to curb contamination. In a study, Tomov et al. performed a review aimed at gauging the impact of a new surgical-site-infection (SSI) prevention bundle in their spine service. The bundle paired a 0.3% Betadine wound rinse with a one-gram dose of intrawound vancomycin powder. To tease out patient-level predictors of infection, the team applied mixed-effect logistic regression. Following implementation, the SSI rate slumped by half. The share of methicillin-resistant Staphylococcus aureus plunged from 30% to 7%, while multibacterial infection rates fell from 37% to 27%. The authors observed that pairing Betadine wound irrigation with intrawound vancomycin powder cut surgical-site infection rates in half—a decline that was both clinically evident and statistically significant. Bacteriologic analysis and risk-factor assessment proved useful for gauging the prophylactic measure’s efficacy and for shaping future protocols [82]. Nonetheless, the concentration of antiseptic solutions warrants careful attention: povidone–iodine concentrations above 1% can impede healing by exerting cytotoxic effects on fibroblasts and other reparative tissues. Mu et al. carried out a meta-analysis to see if wound irrigation with povidone–iodine during spine surgery actually cuts the rate of postoperative surgical site infections. Their combined cohort included 6777 patients. The pooled results showed a significant difference in SSI incidence, between the povidone–iodine-treated group and the control group. Patients who received wound irrigation (IOWI) together with PI experienced fewer deep and superficial infections after spine surgery than those in the control cohort (superficial infection: RR = 0.28, 95% CI = 0.14–0.54; deep infection: RR = 0.24, 95% CI = 0.10–0.60). Sensitivity analyses reinforced the robustness of these findings. This was underscored by strong evidence, after consolidating the data on overall postoperative SSI rates and the incidence of both deep and superficial infections. Utilizing wound irrigation with a povidone–iodine solution during spinal surgery seems to noticeably lower the rate of postoperative surgical site infections [83]. Table 1 shows intraoperative vancomycin powder dosing. The other studies confirmed at the long-term clinical follow up of a low incidence of infection, which provided additional evidence for the benefit of antimicrobial techniques and risk factor mitigation for the prevention of SSI in AIS and young adult Idiopathic Scoliosis surgery [84].

Topical vancomycin is potentially considered as an effective adjunct in preventing SSIs in both spinal and thoracic surgeries. Nevertheless, the newest meta-analytic work has failed to corroborate claims about the efficacy of topical vancomycin. A recent meta-analysis, which pooled seven randomized controlled trials encompassing 2235 patients, assigned 1095 of them (approximately 49%) to receive intrawound vancomycin during open spine surgery. In that cohort, the overall incidence of deep surgical-site infections was 3.38% in the vancomycin group compared with 4.08% in the control group. Overall, deep infections were observed in 2.5% of the treatment cohort and 1.8% of the control cohort while superficial infection rates were 0.9% versus 1.8%, respectively. In a random-effects analysis, intra-operative vancomycin showed no association with surgical-site infection rates. When the data were sliced to focus on patients who underwent spine surgery, vancomycin did not show a statistically significant link to lower rates of surgical-site infections or deep infections, nor did it raise the odds of Gram-negative or culture-negative infections. Likewise, administering vancomycin intra-operatively did not appear to cut the incidence of either deep SSI in those having open spine procedures. Because of these findings, the utility of intra-operative vancomycin in open spine surgery still calls for validation in larger randomized controlled trials [85]. However, clinicians must balance its efficacy with safety concerns—especially regarding systemic absorption and renal toxicity. With cautious patient selection and dose adjustment, vancomycin powder can be safely integrated into perioperative protocols, but further standardization is needed [66,74,75,76,86,87]. When vancomycin was applied directly into the wound, it markedly lowered both superficial surgical-site infections in instrumented spinal surgeries. In contrast, a 1.3 g/L povidone–iodine solution placed intrawound failed to reduce infections, though it did modestly cut the incidence of superficial SSIs [88,89,90,91].

### 3.6. Protocol Integration and Quality Improvement

Integrating antibiotic use into comprehensive SSI prevention bundles—each designed to tackle several risk factors concurrently—yields markedly better clinical outcomes than relying on single isolated measures [33]. A case in point is the ten-step protocol examined by Elgafy et al. which sought to curb spinal wound infections. Their study demonstrated that bundled care, comprising standardized prophylaxis, meticulous skin preparation and vigilant postoperative monitoring, significantly improved infection rates [33]. Similarly, Bagga et al. examined a care bundle in a sizable cohort of 9607 patients and uncovered a substantial decline in SSI rates when a suite of interventions was applied together [92]. A solid SSI-prevention bundle generally hinges on a handful of components. One staple is preoperative skin decolonization often performed with chlorhexidine–gluconate baths to trim down the burden on the skin. Another step involves screening for Staphylococcus aureus (MRSA) and then applying targeted decolonization protocols, which helps cut the infection odds for carriers. Keeping patients normothermic—defined as a core temperature, above 36 °C during the period—also proves essential as it bolsters immune function and promotes wound healing. In addition, enforcing glycemic control—keeping perioperative blood glucose under 180 mg/dL—correlates with lower infection rates. Providing counseling and steadfast support for smoking cessation also proves essential as smoking is a well-known modifiable risk factor that impairs wound healing. Finally ensuring nutritional support, particularly for malnourished patients, is crucial for optimal postoperative recovery and infection prevention. Turning these bundles into practice hinges on an unwavering institutional pledge, a close-knit multidisciplinary partnership and a sweeping quality-improvement scaffolding. The programs that really take off are usually built on the synchronized choreography of surgeons, anesthesiologists, nurses, pharmacists and infection-control experts all pulling in the direction to lock down standard streamlined care pathways. Hospitals are urged to forge locally crafted protocols that echo the makeup of their cohorts, the regional pathogen tableau, the prevailing antimicrobial-resistance currents and each patient’s singular risk profile [89,90,91,93]. Beyond that, maintaining oversight, carrying out periodic audits, and embedding feedback loops are core to these initiatives. They do not only keep the program aligned with established protocols, but also drive a steady uplift in clinical outcomes.

## 4. Operative Risk Factors and Their Correlation with SSI

Surgical site infections arise from a mix of a patient’s own biology, the virulence of the microbes, and the conditions of the operating room. While patient-related risks such as diabetes, obesity and immunosuppression are well known, the factors that play out during surgery itself are a potentially modifiable piece of the SSI puzzle. Grasping how these elements interact is essential for crafting targeted prevention strategies [4,29,67,68,94].

### 4.1. Duration of Surgery

The interplay between the length of a case and the propensity for an SSI stands out as a perennially documented correlation in the annals of operative research. Prolonged operative windows translate into exposure, to ambient pathogens-sustained tissue hypoxia and amplified soft-tissue insult. Such sequelae frequently accompany complex procedures, thereby compounding the infection threat. Seen through a lens, the longer a surgery drags on, the more routes open up for bacteria to slip in. Each extra minute in the theater translates into a cloud of airborne particles and a heightened chance that repeatedly handled instruments or supplies become contaminated.

Extended procedures also grant technique more opportunities to falter. Meanwhile, tissue concentrations of antibiotics wane as the clock ticks and the prolonged exposure promotes tissue desiccation and reduced blood flow—both of which sabotage healing and blunt immune defenses. Notably, the tie between duration and infection risk does not follow a neat linear trend; it ramps up exponentially. After crossing time benchmarks, the infection probability spikes dramatically. Kim et al. [94] examined a cohort of 4588 patients who underwent lumbar fusion for chronic back pain. Their multivariate, risk-adjusted regression models showed that longer operative times were linked to a step-wise rise in the odds of any complication, as well as in medical complications, surgical complications, superficial surgical-site infections and postoperative transfusions. When a lumbar fusion drags on for five hours or more, patients face a higher risk of several complications—another operation organ/space infections at the surgical site, sepsis or septic shock wound dehiscence and even deep-vein thrombosis. That makes length more than just a statistic; it is a pivotal quality metric for these procedures. To improve outcomes, it is essential to find ways to shave off time and to investigate the factors that tend to prolong surgery [94]. A handful of studies indicate that once the clock passes the six-hour threshold, the risk curve sharpens dramatically with the chance of infection climbing to four or five times that of shorter surgeries. Shen et al. [2] set out to determine which variables might foretell delayed infections in scoliosis cases. Within a pool of 3463 patients who received surgery, 17 were flagged as having such late-onset infections. The authors inferred that these delayed infections likely arise from a web of intertwined factors with duration and the administration of allogenic blood transfusions standing out as the primary drivers [2]. In a study by Algarny et al. [37] that evaluated 75 patients, a significant correlation was found between the length of the surgical procedure and the pathogens identified postoperatively. Longer operative times were linked to species of bacteria responsible for postoperative SSI. Accordingly, the use of antibiotics—whether longer-acting agents or repeated intravenous prophylactic doses—should be further assessed [37]. SSIs are tied to a multitude of risk elements, some rooted in characteristics and others in the operative technique. In this review they succeeded in isolating risk variables—both those amenable to intervention and those that are fixed—information that proves indispensable for surgical strategizing and patient dialog [95,96].

These observations carry weight for both the blueprint laid out before the incision and the choices made once the patient is on the table. Surgeons have a toolbox of tactics to lower SSI odds, chief among them trimming the clock on time. When a case swells in scope or complexity, breaking it into staged procedures can prove advantageous. Sharpening the flow of the operating room—through team choreography and clearer dialog—often trims the minutes as well. A meticulous preoperative plan, complete, with a review of imaging and a well-stocked organized set of instruments helps sidestep delays once the surgery gets underway. In the investigation by Cvijanovic et al. [11], the researchers sought to ascertain the incidence rate (IR). They pinpoint risk factors for surgical site infection (SSI) among individuals undergoing thoracic surgical procedures. Across a window of hospital monitoring they prospectively tracked SSI occurrences. Those who experienced an SSI were contrasted with those who did not. They operated on 3370 patients and 205 of them—about 6.1%—developed a surgical-site infection. The infections split into 190 after open thoracic surgery and 15 following video-assisted thoracic procedures. Five independent risk factors for SSI emerged, wound contamination, the length of drainage, patient age, and the duration of the operation [11].

When an operation is expected to be lengthy, bolstering measures—such as fine-tuning antibiotic timing and rigorously managing re-dosing cycles—becomes especially important. Together, these observations underline the necessity of controlling operative duration, a vital component of a larger plan to diminish postoperative infections and boost patient outcomes.

### 4.2. Intraoperative Blood Loss and Transfusion

Excessive intraoperative blood loss is a well-recognized and multifactorial risk factor for SSIs. Its impact extends beyond simple volume loss and involves a complex interplay of hemodynamic, immunological, and local tissue effects. This association has been consistently observed across multiple surgical fields, with particularly strong evidence emerging from spinal and thoracic surgery [54].

From a pathophysiological perspective, blood loss increases infection risk through several interconnected mechanisms. When substantial blood loss occurs, tissue oxygen delivery is significantly impaired, which in turn reduces the oxidative burst activity of neutrophils and other immune cells—an essential function in the body’s early defense against infection. In addition to this hypoxic environment, both acute blood loss and the subsequent need for transfusion have been shown to suppress the immune response. This immune suppression may occur through multiple mechanisms, further reducing the body’s ability to fight off pathogens. Furthermore, massive blood loss can contribute to dilutional coagulopathy, which compromises both hemostasis and proper wound healing. Hemodynamic instability, often manifesting as hypotension, adds another layer of risk by reducing perfusion to the wound margins, further impairing tissue recovery. In the study conducted by Shen et al. [2], the investigators set out to tease the predictors of delayed postoperative infections in scoliosis correction. Among a cohort of 3463 operated individuals, only 17 eventually manifested these late-onset infections. Their analysis suggested that such infections are probably the product of an interplay of factors, most prominently the duration of the operation and the administration of allogenic blood transfusion. Patients above 68 years old, with surgical durations exceeding 156 min, intraoperative blood losses surpassing 475 mL, those with diabetes, and smokers are at significant risk for SSI after lumbar surgery [97].

Quantitative thresholds of blood loss have been studied extensively, revealing specific volumes that correlate with elevated SSI risk. For instance, the studies reported that losing more than 500 mL of blood during spinal procedures more than doubled the likelihood of developing an SSI [32,33,54]. Other investigations have suggested that blood losses surpassing 1000 mL may represent a critical point at which immune function is significantly compromised.

Another layer of complexity comes from transfusion-related immunomodulation (TRIM), which describes the immunosuppressive effects following the administration of allogeneic blood. Transfusion can independently increase the risk of postoperative infections. It has been linked to decreased lymphocyte proliferation, reduced natural killer (NK) cell activity, altered cytokine production, and heightened regulatory T-cell responses. Notably, these immunomodulatory effects may persist for days or even weeks after the transfusion, creating a vulnerable window during which patients are more susceptible to infection.

Given the strong evidence connecting blood loss to postoperative infection, modern surgical practice increasingly emphasizes blood conservation strategies. These efforts begin well before the patient enters the operating room. Preoperatively, optimizing hemoglobin levels through iron supplementation, erythropoietin therapy in selected patients, and—when appropriate—autologous blood donation can reduce the likelihood of transfusion. During surgery, meticulous surgical technique aimed at achieving optimal hemostasis is essential. The intraoperative use of topical hemostatic agents, administration of tranexamic acid, and implementation of controlled hypotension (when safe and indicated) can all help minimize blood loss. Cell salvage technology may also play a beneficial role in reducing the need for allogeneic transfusions [54].

In the postoperative phase, applying restrictive transfusion thresholds has proven effective in reducing unnecessary exposure to transfused blood. Continued iron supplementation and careful monitoring for signs of delayed bleeding further contribute to overall blood management. Together, these strategies form a comprehensive approach to minimizing both blood loss and the associated risk of SSIs, thereby improving surgical outcomes and supporting patient recovery.

### 4.3. Surgical Extent and Instrumentation

The more intricate and expansive a surgery becomes, the higher the likelihood of a surgical-site infection—larger operations typically involve greater tissue trauma, the placement of foreign material, and longer time under the lights. Surgical site infection (SSI) risk is determined not only by surgical approach but also by procedural complexity of operation as well as anatomical region of operation [98,99]. It might obscure significant differences within subgroups to classify all spine or thoracic operations as a single group. Sorting out approaches, especially the choice between anterior and posterior spinal operations or between an open thoracotomy and a thoracoscopic thoracic procedure—turns out to be a pivotal factor when trying to gauge the risk of surgical-site infection (SSI). Each technique brings its blend of tissue trauma exposure to environmental contaminants and hurdles in achieving solid hemostasis and reliable closure. Pointing out how infection risk shifts with the chosen approach is therefore worthwhile. Grasping the impact of the route on SSI rates is essential, for fine-tuning prophylactic measures and ultimately improving patient outcomes. Surgical approach stratification— the distinction between anterior versus posterior routes in spinal operations and thoracotomy versus thoracoscopic (VATS) techniques in chest surgery—yields a refined perspective, on infection risk grounded in the anatomical, procedural and microbiological disparities. This pattern is especially pronounced in procedures where each additional fused level and every extra piece of instrumentation correspond to a rise in infection rates. Multilevel spinal fusion surgeries reveal a dose–response trend for surgical site infections (SSI). With a single level fused, the baseline infection odds hover between one and three percent. Once the scope widens to two or three levels, the risk nudges upward to three to six percent. If the construct spans four or more levels, the SSI frequency can breach the eight-to-twelve percent window. The upward swing in these procedures stems from interrelated reasons [61,82,100,101,102]. Larger incisions are now the norm, exposing tissue while the surgeries drag on longer and involve more blood loss [54]. In addition, they typically demand extensive dissection and devascularization of the tissue and often call for larger or a greater number of implants—each factor nudging the infection risk higher [25,101,103,104,105,106]. Spinal instrumentation, though a linchpin for restoring the spine’s stability, also drags along a suite of infection-related headaches. The glaring issue is the propensity for biofilm development. Bugs like Staphylococcus epidermidis and Staphylococcus aureus love to hitch a ride on metal surfaces, weaving biofilm matrices that shrug off antibiotics and dodge the body’s immune patrols. On top of that, simply having an implant in the body can spark an inflammatory reaction that dulls the local immune defenses. When the hardware fails to meld with bone—or simply loosens—it can carve out voids that become little doorways for bacteria to set up shop. In revision surgeries, the chance of a surgical site infection climbs higher [10,27,98,99,107]. These procedures often require working through tissue planes that have already been disturbed and a blood supply that is compromised which can markedly impede wound healing. The scar tissue— abundant in revision cases—has a modest capacity for regeneration and typically reacts poorly to further trauma. There is also the danger that infected or colonized material may remain in place. Moreover, such operations tend to last and involve greater blood loss, both of which blunt the local immune response. The anterior approach to surgical spine procedures as well as the posterior approach varies extensively regarding anatomical exposure, surgical time, as well as tissue damage that basically determine SSI risk. Several research works proved that posterior approach is riskier for SSIs than anterior approach. Posterior course involves greater dissection of the muscles that result in wider damage to the soft issues, increased duration of exposure to the wound as well as increased usage of instruments which overall enhance susceptibility to infections [108]. The posterior spinal field is closer to skin flora such as Staphylococcus aureus and Cutibacterium acnes, which are the primary causative agents in spinal SSIs. The anterior approach bypasses much of this exposure and thus benefits from a comparatively cleaner field [109]. A total of 11,401 patients undergoing spine surgery for thoracic and lumbar fractures were investigated in the work in Ref. [47], whereas 860 of these patients developed SSI (7.5%). Risk factors significantly associated with SSI in univariate analysis included diabetes, obesity, and increased age. The anterior approach commonly entails a retroperitoneal or transabdominal course to reach the spine with minimal incision and muscular dissection. Some research presented markedly decreased infection rates in anterior cervical discectomy and fusion (ACDF) than in posterior cervical operations [103]. Minimal exposure to dirty fields as well as rapid healing of wounds might be responsible for this benefit. The utilization of minimally invasive surgery (MIS) in the lower back spine decreased infection significantly. The management of this complex pathology may be better addressed via traditional open surgery. In a subgroup comparison, the complication rate in the open in situ fusion (36.3%) versus open reduction (20.6%) subgroup was non-significant. However, the total complication rate in the MIS reduction group (55%) was significantly higher than MIS in situ fusion [104]. In lumbar spine surgery, MIS TLIF produces comparable clinical and radiologic outcomes to open TLIF with the benefits of decreased intraoperative blood losses, shorter operative times, shorter hospital stays, and fewer deep wound infections [105]. Traditional open thoracotomy is associated with significantly higher rates of wound complications and SSIs due to its invasive nature. Muscle splitting, rib spreading, and large incisions increase tissue trauma and local inflammation, which delay wound healing and elevate infection risk [14]. The findings suggest that VATS is associated with a lower risk of postoperative wound infections compared to open thoracotomy, which has implications for surgical decision-making in lung cancer treatment. In a large cohort including 722 patients [110], 392 patients (54.3%) underwent an open thoracic operation, 259 patients (35.9%) underwent VATS surgery, and 71 patients (9.8%) underwent a robotic procedure. Comparing these surgical approaches, there was no statistically significant difference in the overall incidence of postoperative complications as well as the incidence of wound infections, arrhythmias, prolonged air leaks, respiratory failure, or ICU readmissions. Patients who had a VATS procedure were less likely to develop a postoperative chest infection. VATS lobectomy was associated with a lower incidence of postoperative chest infections [110]. In another study [111], patients who experienced postoperative surgical site infection (SSI) at the chest tube drainage site (CDS) after thoracotomy were analyzed. The postoperative drainage period was 2–15 days. Bacterial species were detected in secretions in 18 of 99 cases (18.2%). Older age was a risk factor for the detection of bacteria at the timing of chest tube removal. Eighteen cases (18.2%) were diagnosed with a presence of SSI at the CDS at the timing of staple or suture removal. Eleven of 18 SSI patients showed delayed wound healing. A higher level of HbA1c was found in patients with delayed wound healing. Enterococcus faecalis infection may influence the development of complex SSI [111]. Minimally invasive thoracoscopic surgeries (VATS) have transformed thoracic surgeries with reduced trauma from surgeries. The minimal cuttings and lower manipulation of lung parenchymas and intercostal muscles led to fast recovery and markedly reduced SSIs. Minimally invasive lobectomy was associated with decreased morbidity and length of stay [112]. The study was to compare outcomes and hospitalization costs among patients undergoing open, video-assisted thoracoscopic surgery (VATS) and RATS lobectomy. In this study [112], out of 15,038 patients, 8501 (56.5%), 4608 (30.7%), and 1929 (12.8%) underwent open, VATS, and RATS lobectomy, respectively. Robotic-assisted lobectomies comprised less than 1% of total lobectomy volume in 2008 and grew to 25% of lobectomy volume by 2014. Both VATS and RATS lobectomies were associated with decreased in-hospital mortality compared to thoracotomy. Minimally invasive approaches were associated with improved clinical outcomes compared with open lobectomy. However, only robotic-assisted lobectomy has had rapid growth in utilization. Despite additional cost, RATS lobectomy appears to provide a viable minimally invasive alternative for general thoracic procedures. There were no noted differences in SSI after surgical treatment by all these methods [112]. Beyond lowering infection risk, minimally invasive approaches tend to shorten hospital stays, ease postoperative pain, and curb opioid use, all of which help the wound heal more smoothly. Equally noteworthy is the reduced time a chest drain remains in place, a known risk factor for surgical-site infection, which stands out as another perk of VATS. Moreover, several studies indicate that in selected patients undergoing thoracoscopic lobectomy, a chest tube may be unnecessary. An invasive approach paired with careful resection underpins the operation promoting rapid convalescence and efficiently averting chest-tube complications [113]. Open thoracic surgeries carry a risk of contamination by both skin flora and nosocomial organisms largely because they take longer and leave the wound exposed for an extended period [63]. In contrast, video-assisted thoracoscopic surgery (VATS) limits exposure. Therefore, it tends to attract fewer resistant microbes such as MRSA. In study [73], the investigators examined the incidence of surgical-site infections (SSI). They explored the associated risk factors among patients undergoing lung resections (LR). In this series, the surgical site infection (SSI) rate after conventional LR reached 14.3%, while the 30-day operative mortality was 1.2%, most deaths being due to pneumonia. When LR was performed via VATS, the SSI incidence fell to 5.5%. Moreover, SSI correlated with operative times, lower serum albumin, the presence of comorbidities, older age, and reduced FEV_1_ [71]. The rise in invasive surgical (MIS) techniques has opened up a compelling alternative in selected situations. By tempering the amount of trauma, these approaches have been linked to a lower SSI risk. Tiny incisions spare tissue, which consequently trims operative times and diminishes blood loss. Those who undergo MIS tend to enjoy a postoperative bounce-back, get up on their feet sooner, and encounter fewer wound-related complications overall. Still, it is essential to recognize that minimally invasive methods are not the fit for every case. When spinal deformities are especially intricate or a surgery requires decompression, the traditional open approach often remains necessary. By classifying surgical methods, physicians may differentiate patients with greater risk of SSIs and adopt personalized prophylaxis methods like administration of topical antibiotics such as vancomycin powder during posterior spine or thoracotomy operations, prolonged systemic antibiotics for open thoracic operations with suspected long operations, and preferred minimally invasive approach (VATS or anterior spine) in high-risk patients with diabetes, obesity, or immunosuppression. Furthermore, approach-based stratification facilitates more accurate benchmarking of quality measures since conjoining anterior with posterior outcomes can mute genuine risk profiles. Stratification of surgical approach is a significant factor in defining SSI risk. Spinal as well as thoracic surgeries with anterior and minimally invasive approaches always yield lowered infection compared with posterior and open methods. This is due to differences in tissue trauma, duration of exposure, microbiology of the wound, as well as complexity of procedure. Understanding these elements and integrating them within clinical practice makes improved infection control methods as well as personalized patient therapy possible.

Spinal fusion of the cervix usually requires smaller incisions, reduced dissection of soft tissue, and reduced operative time than does lumbar fusion. Spinal fusions of the cervix, particularly anterior cervical discectomy and fusion (ACDF), are commonly carried out with minimally invasive methods and are less instrumented. Conversely, posterior interbody fusion of the lumbar spine (PLIF) or transforaminal interbody fusion of the lumbar spine (TLIF) usually requires significant dissection of the muscle, larger implants, and greater exposure. These technical and anatomical variations have immediate postoperative infection risk implications. Several large-scale reviews have indicated that lumbar fusion possesses a significantly greater SSI prevalence than cervical fusion. Invasive surgery (MIS) appears capable of trimming the rate of surgical site infection (SSI). A retrospective review of 1352 patients who together underwent 1382 lumbar fusion operations showed that the open approach was the most common (39.3%, 543 cases), followed by mini-open (33.1%, 458), and MIS (27.6%, 381). Across the cohort only 0.94% of patients developed an SSI after a one- or two-level posterior lumbar instrumented fusion. Although the cohort’s observed SSI rate was about fourfold that of the MIS cohort, the paucity of cases left us unable to extract a statistically significant gap among the three surgical approaches [106]. In another study [101], researchers carried out a meta-analysis to pinpoint risk factors for SSI after surgery. They found an SSI incidence of 2.9% (1222 infections among 41,624 procedures). The analysis linked variables to a higher SSI risk: the fusion approach (anterior versus posterior and anterior versus combined), the use of osteotomy intra-operative blood transfusion, a history of diabetes or prior surgery, hypertension, the anatomical region operated on (cervical versus thoracic; lumbar, versus thoracic), underlying osteoporosis, and the number of vertebral levels fused. Nonetheless age, sex, smoking history, body-mass index, fusion approach (posterior versus combined), surgical level (cervical, versus lumbar), operative duration, blood loss, steroid use, dural tears, and albumin levels were not linked to the development of SSI [101]. The authors of study [102] examined SSI as a complication after spine surgery, emphasizing its association with notable morbidity, poorer clinical outcomes, and increased healthcare costs. They carried out a cohort study of dSSI in adult patients who underwent posterior cervical spine surgery at ten research hospitals. Among the 2184 adults enrolled, 28 (1.3%) experienced a postoperative deep SSI. Multivariable regression analysis identified two risk factors that were statistically significant: occipitocervical surgery and male sex. The subgroup analysis revealed surgery as the lone independent risk factor for deep surgical-site infection in patients who underwent instrumented fusion. Because occipitocervical surgery is relatively uncommon, the investigators relied on a multicenter cohort to support this observation [102]. The heightened risk in lumbar cases might be due to broader surgical fields and greater postoperative blood loss, longer surgical times, increased hardware usage and implant usage, and greater revision operation likelihoods. In addition, patients who undergo lumbar fusion are frequently presenting with comorbidities such as obesity, diabetes, or history of smoking that add to the base-line infective risk [54]. Although both are significant thoracic surgeries, esophagectomy is significantly greater in complexity than lobectomy and entails two to three surgical sites (abdominal, thoracic, and occasionally cervical).

In the study of Tsantes et al. [1], the authors set out to probe whether nutritional deficits constitute a risk factor for surgical-site infection after spinal surgery. They considered cohort and case–control investigations that examined malnutrition as a predictor of SSI in patients undergoing procedures. Altogether, 22 studies encompassing more than 175,000 participants—roughly 2.14% of whom experienced a postoperative SSI—were reviewed and the analysis revealed that infections were indeed more frequent in malnourished patients. Even though patients who were malnourished before surgery faced a higher risk of surgical-site infection, after thoracolumbar or sacral spine procedures this link did not appear in those undergoing cervical spine surgery. The research of Gupta and team [16] set out to explore whether the spine adipose index is linked to surgical-site infections and to pin down a cutoff point that could aid preoperative risk stratification for patients undergoing posterior instrumented lumbar fusion (PILF). They identified every patient who experienced a primary incisional or organ-space SSI within 90 days after surgery applying the CDC’s criteria. For each of these cases, they compiled a set of potential preoperative and intra-operative risk factors for deep infection. The final analysis encompassed 42 patients—21 cases and an equal number of matched controls. In those who subsequently developed a surgical-site infection, the spine adipose index was conspicuously higher, and this association persisted after adjustment for confounders. A spine adipose index of ≥0.51 corresponded to a two-fold increase in the risk of deep SSI after PILF surgery. Reliability of the spine adipose index was excellent, with both inter- and intra-observer intraclass correlation coefficients exceeding 0.9 (*p* < 0.001). In the realm of metrics, the spine adipose index has stepped onto the stage as a fresh measure that independently flags the odds of a deep surgical site infection; a threshold of 0.51 seems to provide the most reliable preoperative risk stratification for patients undergoing PILF surgery. With obesity on the rise, determining how it affects 30-day readmissions and patients’ own sense of health is crucial for proper risk stratification. The authors set out to determine whether obesity alone predicts 30-day readmissions after elective spine surgery. The research team pored over the records of 500 patients undergoing elective spine surgery at a major academic medical center. The authors found that obesity before surgery is, on its own, a predictor of a readmission within 30 days after a spine operation. In a healthcare climate that is increasingly cost-conscious, a patient’s preoperative BMI can flag those who are likely to experience an early unplanned hospital readmission [15]. In similar studies for a pediatric population, the researchers [8] set out to pinpoint risk factors for surgical-site infections after pediatric spinal fusion. In a university-hospital series of 598 children who underwent the procedure, 22 developed an SSI. They applied both multivariable statistical models to tease out which variables were linked to infection and to gauge the infections’ outcomes. Their analysis suggests that extending prophylaxis to cover Gram-negative organisms could be beneficial for these patients. Moreover, the length of the operation, the use of graft implantation, and the amount of blood loss emerged as operative factors that might be adjusted to lower infection risk. Neuromuscular scoliosis, an above-average weight-for-age, and an ASA classification of three or higher can signal to the team that a patient is at heightened risk for a surgical site infection.

Lobectomy is a single-site procedure that is more commonly carried out with video-assisted thoracoscopic surgery (VATS). Invasiveness and risk of contamination due to esophagectomy render it per se more prone to infections such as anastomotic leaks, mediastinitis, as well as deep wound infections. Hybrid invasive esophagectomy (HMIE) has emerged as the preferable alternative to traditional open esophagectomy showing a clear cut-back in postoperative morbidity [72]. In the hybrid technique, the conventional laparotomy is replaced by a minimally invasive laparoscopic approach. The comprehensive mediastinal resection and subsequent reconstruction are still carried out via a thoracic route using a muscle-sparing thoracotomy. Nonetheless, 54% of patients experienced at least one perioperative complication. An anastomotic leak was noted in 1.9% of the patients and a single individual experienced focal conduit necrosis of the gastric pull-up. Post-operative pulmonary morbidity affected 31% of the cohort with pneumonia diagnosed in 17%. The 90-day mortality stood at 2.5%. Wound infection occurred in 3% of cases. Delayed gastric emptying was observed in 17% of the patients [72].

The elevated risk of infection in esophagectomy patients is reinforced by preoperative malnutrition, immunosuppression secondary to cancer or chemotherapy, a long operative time, and gastrointestinal content contamination. Lobectomy, on the other hand, is commonly undertaken in patients with localized disease of the lung with intact nutritional reserves so that patients’ preoperative risk is lowered at base level. Minimally invasive esophagectomy (MIE) and VATS, and even lobectomies performed through invasive routes appear to sidestep many of the complications that trouble their open-surgery counterparts. The newest analysis [114] demonstrates that invasive esophagectomy (MIE) cuts down on wound-related problems yet deep infections such as mediastinitis remain fairly common often because of leaks at the anastomosis. Of a total of 2550 patients, 1218 (47.8%) underwent MIE while 1332 (52.2%) received esophagectomy (OE). After adjusting for risk, MIE was linked to an odd of postoperative complications. In the subgroup analyses, the in-hospital mortality fell appreciably for patients who received MIE. Hence, forthcoming practice and infection-control directives should embed these procedural distinctions rather than cling to sweeping generalized policies. Table 2 shows the risk stratification framework for SSI Prevention.

### 4.4. Synergistic Risk Profiles and Predictive Modeling

The operative risk factors discussed earlier do not act in a vacuum; they intertwine in a synergistic dance shaping a patient’s overall odds of developing a surgical site infection. Unraveling these threads is essential for pinning down a nuanced risk assessment and for crafting prevention strategies that are both more precise and more effective. Across clinical settings, the way several risk factors stack up often drives infection odds well above what a simple addition would predict—their effects tend to multiply. For example, a patient undergoing a spinal fusion that spans three or more levels with a surgery lasting more than four hours and blood loss exceeding 500 mL may confront an estimated surgical-site infection risk of roughly 12% to 15% [4,9,10,47,103,104,105,107,115]. In a meta-analysis conducted by Zhang et al., the reported incidence of surgical site infection (SSI) after spinal surgery was 2.9% (1222 of 41,624) and the analysis also linked several variables—fusion approach (anterior versus posterior; anterior versus combined) osteotomy, transfusion, a history of diabetes and prior surgery, hypertension, surgical location (cervical versus thoracic; lumbar versus thoracic) osteoporosis, and the number of fusion levels—with a higher likelihood of SSI after spinal surgery. Nonetheless, age, sex, smoking history, body-mass index, the fusion approach (posterior versus combined) the surgical region (cervical versus lumbar), operative time, blood loss, steroid use, dural tears, and albumin were all unrelated to the development of SSI [101]. In contrast, a peer patient with a solitary risk element—such as a lengthened operative duration—could be staring at a dramatically reduced chance hovering in the ballpark of four to six percent. To precisely gauge and foresee these hazards, a number of validated risk-scoring systems have been created, e.g., the NNIS Risk Index, a staple in hospital folds; the American Society of Anesthesiologists (ASA) physical status score—which determine the wound classification and the length of the operation. A gamut of prediction models exists to gauge the risk of complications after spine surgery. Before any of these can be rolled out in practice, they must undergo validation and head-to-head comparison [116]. Strikingly, none of the models for spine-surgery adverse events have been put through decision-curve analysis. They are the following: SpineSage, the Risk Assessment Tool (RAT) and the National Surgical Quality Improvement Program Risk Calculator (NSQIP), for forecasting 30-day complications following spine procedures. Looking across the models, SpineSage delivered the risk forecasts and should outshine a blanket-treat-everyone approaches whenever a clinician or patient flags a complication risk above ten percent as noteworthy. By contrast, the NSQIP system appears ill-suited for clinical use in our local population [116]. Meanwhile, the SpineSage model—built expressly for procedures—melds patient-centric data with intra-operative variables [117]. Moreover, the ACS NSQIP Risk Calculator delivers risk estimates by drawing on an extensive range of clinical and operative characteristics. The recent surge in modeling is beginning to sharpen clinical decision-making [116,117,118]. These findings highlight just how vital it is to bring integrated risk-assessment tools and predictive models into care. By wrestling with the variables, at play, clinicians can shape more personalized prevention tactics, steer resources where they are needed most and ultimately ease the load of postoperative infections [119].

### 4.5. Clinical Decision-Making

Predictive decision-making tools have become a cornerstone of today’s practice. By feeding risk numbers into pre-op counseling, they give patients a more realistic sense of their own odds. The same models also steer measures toward those who need them most, so high-risk individuals receive a heightened level of care. Beyond that, they act as a triage compass for resources nudging interventions to the patients where they will make the biggest difference. Moreover, they are pivotal to the push for quality improvement granting the ability to monitor outcomes more precisely and to spot where the system as a whole could be fine-tuned. Melding a host of patient-specific factors into robust risk-assessment frameworks marks a noteworthy stride toward customizing SSI prevention. By aggregating insights on complexity, patient comorbidities, and procedural nuances, these tools enable clinicians to fine-tune preventive strategies more precisely than ever before.

## 5. Negative-Pressure Wound Therapy (NPWT)

NPWT, sometimes referred to as vacuum-assisted closure (VAC), has taken its place as a game-changer in wound care. Rather than waiting for a wound to deteriorate and then treating it, the approach leans toward preventing trouble before it starts, especially in high-risk surgical procedures. Over the decades, its use in thoracic and spinal operations has surged, and an ever-growing body of data backs up its ability to slash surgical-site infection rates and push wounds toward faster, cleaner healing [120].

### 5.1. Mechanism of Action and Physiological Effects

NPWT clamps a sealed dressing onto the wound. It links it to a vacuum pump applying a precisely controlled sub-atmospheric pull to the wound bed. The resulting cascade of multimodal therapeutic actions interlocks forging a near-ideal milieu for tissue repair while simultaneously dampening the chance of infection. Negative-pressure wound therapy dramatically lowered infection rates in patients burdened with comorbidities, hinting it could be a valuable tool for handling wound infections in that subgroup. The result is especially pertinent to minority populations underscoring the need for customized wound-care strategies [121].

Enhanced perfusion and angiogenesis: The mechanical tension generated by negative pressure therapy prompts cells to proliferate and sprout new vessels through mechano-transduction pathways. This cascade lifts the expression of VEGF and a host of pro-angiogenic mediators, thereby sharpening microvascular flow and enriching tissue oxygen levels. Laser-Doppler flowmetry measurements have recorded perfusion jumps of roughly two- to threefold, in the 24–48 h after NPWT is applied. Edema Reduction and Tissue Approximation: Steady suction draws out the fluid that pools between cells, easing the swelling that would otherwise choke off oxygen diffusion and slow cellular metabolism [122,123]. As tissue pressure drops, the gradients that drive perfusion sharpen, allowing nutrients to reach the healing area efficiently. The mechanical pull also nudges wound edges together, shrinking any pockets that could become a breeding ground for bacteria. Bacterial Clearance and Biofilm Disruption: Continuous drainage keeps wound fluid from stagnating, removing a nutrient-rich environment for microbes. The negative pressure forces can tear apart biofilm layers and improve the infiltration of systemic antibiotics into the wound bed. Research shows bacterial counts can plunge ten- to hundred-fold within 48–72 h of NPWT treatment [121,122,123,124]. Cellular Proliferation and Wound Contraction: Mechanical cues give fibroblasts a boost, prompting them to multiply, lay down collagen, and pull the wound edges together. At the time, a carefully regulated environment keeps moisture at the sweet spot while shielding the wound from outside contaminants, creating an ideal stage for cells to migrate and new tissue to regenerate.

Molecular and Cellular Effects: They are described in recent studies which are beginning to peel back the curtain behind the healing boost that NPWT provides. A standout observation is that NPWT ramps up a trio of growth mediators—vascular endothelial growth factor (VEGF), platelet-derived growth factor (PDGF), and fibroblast growth factor (FGF)—each a cornerstone in the cascade that rebuilds tissue and drives repair. In addition, NPWT appears to crank up the activity of matrix metalloproteinases—those enzymes that orchestrate tissue remodeling and wound repair. The therapy also drives up the expression of anti-inflammatory cytokines, which act to temper the cascade and nurture a more supportive healing landscape. Moreover, NPWT lifts performance, sharpening the body’s capacity to wipe out bacteria and cut down the risk of infection at the wound site [124,125,126,127,128].

### 5.2. Evidence Base in Spinal and Thoracic Surgeries

The evidence supporting NPWT use in spinal and thoracic surgeries has grown substantially, with multiple high-quality studies demonstrating significant benefits in SSI reduction and wound-healing outcomes. Ren et al. [127] conducted a database search initially pulling 571 papers into the review. After applying the predefined inclusion criteria, 19 studies remained for analysis. Their meta-analysis showed that the average duration of vacuum sealing drainage (VSD) therapy was 17.45 days with patients receiving VSD roughly 2.57 times on average. The post-discharge infection recurrence rate was low at 2% and the reoperation rate for fixation, among those treated with NPWT, was 4%. There is evidence that NPWT can effectively treat deep surgical-site infections after spinal surgery, pointing to its practical usefulness. A handful of high-quality randomized controlled trials have amassed evidence that negative-pressure wound therapy is effective. Hayakawa et al. [122] chronicled a case of upper-thoracic spondylitis complicated by a paravertebral abscess, which was successfully managed through a blend of NPWT, a cranked-rod construct, and a minimally invasive posterior-only technique. The severity of the patient’s deficits coupled with the abscess prompted a decision for surgical treatment. Nonetheless, the surgeons opted for a route for drainage and debridement in order to spare the adjacent organs from injury. Because the posterior approach only permitted a debridement, during surgery, the team added NPWT to guarantee continuous postoperative drainage. In the end, they reported a cure of primary pyogenic spondylitis with a paravertebral abscess in the upper thoracic spine using posterior instrumentation combined with NPWT. This case shows that when complete removal of tissue cannot be achieved, NPWT can act as a valuable adjunctive treatment. Riding on those results, Martin et al. [123] presented the study’s primary endpoint centered on how long negative pressure could be sustained on the wound over a seven-day span. Healing was evaluated at day 7, and again at day 30. Scar quality was examined on days 14 and 30 alongside assessments of management and overall ease of use. A total of 23 patients took part in the trial. Nine adverse events occurred among five participants. Importantly, the treated wounds were not painful. Participant and clinician assessments indicated that the treatment was straightforward to apply in the hospital and just as easy to manage at home during activities. The study’s authors concluded that the generated incisional negative pressure device performed effectively in clinical practice and proved to be both convenient and user-friendly [123]. Recently, a retrospective review of patients with pressure ulcers who underwent reconstructive surgery from March 2019 through April 2023 [128] was undertaken. The operative protocol consisted of debridement followed by coverage using gluteal-artery-perforator-based fasciocutaneous flaps, after which postoperative monitoring was performed. In the cohort of 52 individuals, those managed with negative pressure wound therapy exhibited a reduction in flap-related complications and a noticeably lower drainage volume on the seventh postoperative day compared with the group receiving conventional dressings. The NPWT cohort showed no complications whereas the conventional cohort (*n* = 27) recorded one infection and three instances of wound dehiscence. Nonetheless, flap survival was perfect, in both groups reaching a 100% rate. In the investigation documented as [128], the investigators reported that employing closed-incision negative-pressure wound therapy may curtail the incidence of both dehiscence and infection in the elderly cohort undergoing sacral pressure-sore reconstruction with local flaps.

A myriad of observational studies have consistently validated the therapeutic benefits of NPWT in everyday clinical practice. In a single-institution study by Woldesenbet et al., 266 patients who underwent posterior thoracic, lumbar or thoracolumbar spine surgery performed by one neurosurgeon were evaluated. Among them, 122 (46%) were male and 144 (54%) were female. The average age was 56.4 years, and the mean body-mass index was 29.6 kg/m^2^. NPWT was administered to 153 patients accounting for 57.5% of the cohort. In the period, wound infections were recorded in 35 patients—about 13% of the total—and 19 of those cases (roughly 54%) occurred in the NPWT group. A focused look at individuals carrying comorbidities showed a pronounced decline in infection rates when NPWT was applied. The investigators therefore concluded that NPWT markedly reduces infection risk in this high-risk subgroup, suggesting a benefit for managing wound infections among those patients [121]. Imtiaz et al. reported using a PICO closed-incision negative pressure wound-therapy dressing as prophylaxis after spinal surgery, noting a reduction in SSI rates in a single-center retrospective study [120].

### 5.3. Indications for Prophylactic NPWT

The choice to start NPWT should rest on a clear individualized risk assessment that weighs both the patient’s specific characteristics and the details of the procedure. An expanding body of evidence now leans toward a risk-stratified approach pinpointing high-risk situations where NPWT is unmistakably indicated [126,127]. One of the widely recognized reasons to employ prophylactic NPWT is obesity, especially in patients whose body-mass index reaches 35 or higher. Those with obesity contend with a host of hurdles that can undermine wound repair. Among them are reduced tissue perfusion caused by a thick layer of subcutaneous fat, increased mechanical strain on the surgical incision, and a higher incidence of comorbidities such as diabetes. Together, these factors raise the likelihood of wound dehiscence and infection. In their report, Chen et al. showed that NPWT offers marked benefits for this cohort, underscoring its preventive value [129]. People with diabetes mellitus also belong to a high-risk group for surgical-site complications. Diabetes hinders healing through a mix of pathways—dysfunction, dampened immune-cell activity, inadequate collagen formation and an overall higher baseline infection rate. Research by Adogwa et al. demonstrates that applying NPWT to patients improves wound outcomes and reduces postoperative infections [130]. Routine use of incisional NPWT was associated with a significant reduction in the incidence of postoperative wound infection and dehiscence. Complex spinal procedures that require instrumentation across three or more levels should prompt a thorough consideration of prophylactic NPWT. Such operations typically involve incisions, extensive tissue disruption, longer surgical times, greater blood loss and a heavier load of implanted hardware—factors that markedly raise the risk of postoperative complications [25]. NPWT seems to knock down the odds of wound dehiscence and surgical-site infection in folks undergoing a lumbar fusion. When the therapy is earmarked for patients vulnerable to postoperative wound problems, it helps keep the incidence of dehiscence and SSI from climbing [125]. Beyond the patient-specific hazards, a handful of variables also raise the odds of a surgical site infection. Procedures that stretch beyond four hours, those with blood loss exceeding 500 mL, hybrid approaches such as spinal fusion, and any revision or surgery performed in a previously irradiated field each create a more hostile wound environment. In that setting, reinforcing wound protection—like applying NPWT—can make a difference. One more factor to consider is the condition of the soft-tissue envelope surrounding the site. Patients with spinal deformities often have tighter skin while those with old surgical scars or skin that is been compromised—perhaps by prolonged steroid use—are especially prone to wound breakdown. In these circumstances, the protective and stabilizing benefits of NPWT can be particularly valuable. New evidence is pointing to a growing role for negative-pressure wound therapy in groups that are not the usual suspects but are just as vulnerable. This spans immunocompromised individuals—recipient patients undergoing chemotherapy—smokers who cannot quit before surgery, and those coping with chronic systemic conditions such as kidney or liver disease. When delayed healing is expected because of underlying local factors, NPWT can serve as a proactive measure, helping to curb complications and promote a smoother recovery.

### 5.4. Technical Considerations and Best Practices

Obtaining the results from negative-pressure wound therapy is not just about picking the right patients—it also calls for careful technique and strict adherence to proven evidence-based protocols. In a meta-analysis [131] that combined eight studies encompassing 1061 patients who had fusion and were managed either with NPWT or with a conventional postoperative dressing, the surgical-site infection rate was markedly lower with NPWT—4.49% compared with 11.32% in the control cohort, a difference that reached statistical significance. A fixed-effects model, however, revealed no disparity between the NPWT group and the standard-dressing group regarding the need for re-operation to perform wound debridement. Furthermore, the incidence of wound dehiscence was comparable between the two cohorts, albeit with a non-significant dip in the NWPT-treated group relative to controls. Although NWPT might meaningfully ease the burden of postoperative wound problems, the meta-analysis did not have sufficient power to substantiate that link [131]. Timing is everything; as soon as primary wound closure is wrapped up, NPWT should be hooked up—ideally while still in the operating room—to lock in sterility and a reliable seal, from the outset. The choice of interface material carries weight. Foam generally wins out because it conforms to wound shapes with ease but when tissue ingrowth looms as a risk, gauze may prove the safer bet. Furthermore silver-laced dressings can supply an antimicrobial boost, which could prove advantageous in wounds that are classified as high-risk. Across clinical studies, the recommended negative pressure falls between –75 and –125 mmHg, with the –125 mmHg setting emerging as the most frequently chosen and the best-supported level for continuous therapy. Arguably the single vital element of NPWT’s efficacy is keeping a tight airtight seal throughout the entire treatment; even a minor loss of seal integrity can undercut the therapeutic benefit. Post-operative therapy generally lasts between three and seven days. Although most guidelines default to a five-day course, the exact length should be individualized for each patient. Factors that shape this decision include the speed of wound healing, the volume and type of drainage, the patient’s comfort level, overall mobility and the risk of complications. Continual oversight is the linchpin for both safety and performance. On a day-to-day basis, clinicians should inspect the seal for any breaches and watch how the drainage behaves. It is imperative that the care team stays alert for signs of infection or skin irritation. Meanwhile, patients need guidance about activity restrictions while using NPWT and a coordinated effort with physical-therapy colleagues can pave the way for safe early mobilization whenever feasible. With its clear benefits, NPWT is not free of hiccups. Seal leaks crop up often—usually because the skin was not prepped properly or body hair gets in the way—forcing a re-application of the dressing. When drainage runs high, the canister may need to be changed more frequently than anticipated. Patient discomfort is another complaint, but a dose of analgesics or a modest reduction in the negative pressure setting often does the trick. Should adhesives provoke skin irritation employing a protective barrier film—or opting for an adhesive system—can shield the skin and sustain patient comfort.

### 5.5. Limitations, Contraindications, and Adverse Effects

NPWT is commonly considered a well-tolerated approach. Still, it remains crucial for clinicians to recognize its boundaries, particular contraindications and any possible adverse reactions in order to apply it appropriately. Absolute contraindications for negative-pressure wound therapy include osteomyelitis, exposure of major blood vessels or internal organs, malignancy residing in the wound bed, an unmanaged active coagulopathy, and fistulas that communicate with internal organs or body cavities. When any of these conditions are present, NPWT poses risk and should be avoided. It is also worth noting that several relative contraindications call for a different approach. Patients on anticoagulant therapy have a risk of bleeding, so their coagulation status needs close surveillance. For some individuals, the noise generated by the NPWT pump can be downright intolerable, potentially compromising their willingness to adhere to the treatment. Additionally, those with mobility often find the device’s physical demands taxing, especially when movement restrictions interfere with walking or hinder rehabilitation efforts. Adverse effects tied to NPWT are relatively rare. They do happen. The side-effects that surface often—showing up in roughly one to five percent of cases—are skin maceration or irritation right at the edge where the adhesive dressing meets the skin. A number of patients also voice discomfort from the pump’s hum or the pulling sensation of suction. Moreover, the device’s nonstop operation can temporarily curb mobility and disrupt sleep. In a small number of cases—about 0.1 to 1%— more serious complications surface. Among these are bleeding episodes, reactions to the adhesive, and occasional infections that stem from a contaminated device. In the exceptional scenario—fewer than 0.1% of patients—far graver outcomes have been documented. These can involve tissue growing into the foam dressing, eventually necessitating extraction; residual foam fragments lodged in the wound; or extensive skin necrosis that calls for urgent medical care. With its occasional downsides, NPWT still delivers substantial upside that boosts patient satisfaction and clinical outcomes. While some patients note discomfort or a temporary limitation in mobility, the bulk of the research consistently points to fewer wound-related complications, faster healing, reduced re-operation rates and a quicker return to everyday activities. Overall, the weight of evidence favors the use of NPWT in carefully chosen high-risk patients undergoing spinal or thoracic surgery. The advantages appear to outweigh the manageable risks, especially when the therapy is applied within established safety guidelines.

## 6. Combination Preventive Strategies for SSIs in Immunosuppressed Patients

SSIs remain a significant source of morbidity, particularly in immunosuppressed patients who exhibit compromised wound healing and attenuated immune responses. These populations include patients with malignancies, autoimmune disorders, those undergoing chemotherapy, transplant recipients, and individuals receiving chronic corticosteroids or biologics. Despite advancements in prophylactic regimens, immunosuppressed individuals continue to experience disproportionately high SSI rates. To bridge this clinical gap, combining topical vancomycin powder (TVP), NPWT, and extended systemic antibiotic prophylaxis presents a promising multimodal approach. Immunosuppressed patients are inherently more susceptible to SSI due to impaired neutrophil activity, reduced cytokine response, and delayed epithelialization. Prophylactic strategies that work in immunocompetent populations may be insufficient for this group, necessitating aggressive and layered approaches. Emerging evidence supports the additive or synergistic potential of combining multiple prophylactic interventions tailored to individual patient risk.

### 6.1. Topical Vancomycin Powder (TVP)

TVP has gained popularity in orthopedic and cardiothoracic surgery, particularly in spine procedures, for its ability to deliver high local antibiotic concentrations directly at the surgical site, minimizing systemic exposure. Mechanism of action: Vancomycin inhibits Gram-positive bacterial cell wall synthesis, including methicillin-resistant Staphylococcus aureus (MRSA), a common SSI pathogen. Evidence in Immunosuppressed Patients: Although data specific to immunosuppressed cohorts are limited, studies have shown that TVP significantly reduces deep SSI rates in high-risk spine surgeries without systemic adverse effects. Luo et al.’s meta-analysis [24] set out to find out whether slathering vancomycin powder (VP) onto the wound could curb site infections (SSIs) after posterior spine surgery. Pulling together the odds ratios reported in the selected studies, the pooled results showed that topical VP does indeed lower the SSI rate in these operations while leaving its effectiveness against infections untouched. Subgroup analysis demonstrated that VP administered at 1 g, 2 g or within the 0.5–2 g range lowered the incidence of surgical site infections. A second subgroup analysis indicated that local VP application—whether after cervical procedures or after thoracolumbar surgery—significantly curtailed the risk of SSIs. Additionally, the proportion of infections caused by organisms including MRSA declined when VP was used intraoperatively, whereas the rate of Gram-negative infections showed no meaningful reduction. The on-site use of VP appears to act as a shield against surgical-site infections, Gram-positive microbes and methicillin-resistant Staphylococcus aureus (MRSA) after a spinal operation [24]. The current literature robustly endorses applying vancomycin powder to incisions, routinely irrigating the wound and swapping gloves often [33]. Nevertheless, the newest meta-analyses have not reproduced claims about the benefit of topical vancomycin. Applying vancomycin during surgery does not appear to lower the incidence of either superficial or deep surgical-site infections in open spine procedures. Consequently, the precise role of vancomycin in this setting remains unresolved and merits further investigation in larger randomized controlled trials [85]. Chlorhexidine wipes for preoperative skin preparation receive backing. Most studies emphasize normalizing HbA1c before surgery to curb infection risk. While limiting flash sterilization is advised, its specific impact on procedures remains unevaluated. Likewise, reducing OR traffic and the number of personnel in the operating theater is recommended, even though Level 1 evidence is still lacking [33]. Clinical Considerations in immunosuppressed patients: the lack of robust systemic immune response places even more importance on local antibacterial defenses, making TVP particularly appealing. Surgical site infection (SSI) that arises after spine tumor resections often forces a postponement of radiation therapy and the start of treatment [132]. In a series of 102 patients who underwent 130 procedures, the overall SSI rate was 3.85%. The tumors were 18.6% primary and 81.4% metastatic. The cohort’s average age was 59.5 years, with 60.8% male and 39.2% female. Preoperative radiation therapy was significantly linked to an SSI risk (*p* = 0.005), while percutaneous instrumentation did not produce a meaningful difference in infection rates. Comparing primary with tumors, the infection rates turned out to be essentially indistinguishable. When a multivariable regression was performed, preoperative radiation emerged as the independent factor that reached statistical significance. The authors [132] point out that preoperative radiation therapy continues to pose a risk for surgical-site infection. In contrast, the use of instrumentation did not translate into higher SSI rates and again no difference surfaced between primary and metastatic lesions. Notably in surgeries where a regimen of Betadine irrigation and intrawound vancomycin powder was employed, the SSI incidence fell to 3.85%.

### 6.2. Negative-Pressure Wound Therapy

NPWT involves applying sub atmospheric pressure to closed incisions, which reduces edema, increases perfusion, and promotes granulation tissue formation. This therapy has shown benefits in high-risk surgical wounds, including obese and diabetic patients. In the investigation by Naylor et al. [125], the authors set out to document a surgeon’s experience with incisional NPWT and to gauge its impact on wound dehiscence and SSIs after instrumented spine surgery. The cohort comprised 393 patients who did not receive NPWT and 76 who did. Midway through the data-collection period, the protocol was adjusted so that every patient undergoing lumbar fusion was treated with NPWT. Among the anterior-lumbar-fusion patients without NPWT, 20% developed an SSI, whereas none of the NPWT-treated patients experienced either complication. In practice, NPWT for spine procedures was applied on a case-by-case basis, guided by the very risk factors that predispose to surgical site infections and wound dehiscence. The NPWT group carried a load of spinal neoplasia, osteomyelitis or discitis durotomies, revision operations, and longer fusion constructs, yet their rates of dehiscence and SSIs were essentially indistinguishable from those of the non-NPWT cohort (5.6% vs. 5.7% *p* = 0.98). The study found that negative-pressure wound therapy reduced both dehiscence and surgical-site infections in patients who underwent lumbar fusion via an anterior approach. When the therapy is applied primarily to those at risk, for postoperative wound complications, it appears to keep the rates of dehiscence and infection from climbing [125]. Similar reductions have been observed across colorectal, vascular, and orthopedic surgeries. Application in Immunosuppressed Patients: Immunosuppressed individuals often have compromised microcirculation and delayed healing. NPWT can mitigate these issues by promoting localized angiogenesis and removing exudate. In spinal fusion surgery, NPWT was associated with reduced infection and faster wound closure. Jeong et al. [128] demonstrated that closed-incision negative-pressure wound therapy holds potential for curbing wound dehiscence and infection rates in patients undergoing sacral pressure-sore reconstruction with local flaps. Caution is warranted in patients with fragile skin integrity or active skin diseases.

### 6.3. Extended Systemic Antibiotic Prophylaxis

Standard antibiotic prophylaxis typically involves a single preoperative dose, occasionally extended to 24 h postoperatively. However, immunosuppressed patients may benefit from prolonged coverage. While the extension of systemic antibiotic prophylaxis beyond 24 h is debated in the general population due to resistance risks, some studies suggest it could be advantageous in immunosuppressed hosts. The lack of immune clearance may justify extended prophylaxis (up to 48 h), especially in surgeries involving implants or prostheses [27,31,33,39,120].

Recommended regimens: Beta-lactam + Glycopeptide (e.g., cefazolin + vancomycin): for MRSA-colonized or high-risk patients. Consideration of local antibiogram and patient-specific flora is crucial [24,25,63,133,134].

### 6.4. Combination Strategy Evidence

While each intervention independently offers protection, the combination may yield additive benefits. Unfortunately, randomized controlled trials specifically investigating the combined use of TVP, NPWT, and extended antibiotics in immunosuppressed patients are lacking. Nonetheless, extrapolation from high-risk subpopulations and theoretical synergy support their joint use. A review by Ren et al. of 571 studies concluded that the current body of evidence backs the effectiveness of negative-pressure wound therapy in handling surgical-site infections after spinal surgery, pointing to its clinical usefulness as well as in various surgeries [26,127,128,135]. Meanwhile, topical vancomycin and extended antibiotics have proven benefits in separate meta-analyses for orthopedic and abdominal surgeries. Based on our analysis, we can construct a risk assessment scale and propose appropriate management strategies, as shown in Table 3.

Immunosuppressed patients remain a vulnerable population for SSIs. Current data support the application of a combined preventive strategy using topical vancomycin, NPWT, and extended antibiotics. While high-quality evidence is limited for this specific patient group, the convergence of mechanistic plausibility and positive outcomes from high-risk subgroups supports this multifaceted approach. Further prospective trials are warranted to validate efficacy, refine timing, and identify optimal combinations in immunosuppressed surgical cohorts [27,31,33,35,39,120,135,136,137,138,139,140,141].

## 7. Outcomes and Complication Rates

The clinical impact of postoperative wound infections following spinal and thoracic surgeries extends far beyond the immediate perioperative period, affecting patient outcomes, healthcare resource utilization, and long-term functional status. Understanding the full spectrum of consequences associated with SSIs is essential for justifying prevention strategies and guiding clinical decision-making.

### 7.1. Clinical Consequences and Patient Impact

How They Affect Patients: SSIs can manifest in an array of forms, each bringing its own set of consequences for how patients recover, the speed of their convalescence, and the nature of any ongoing care they may require. The Centers for Disease Control and Prevention (CDC) classify these infections by depth and tissue involvement, supplying an accepted framework for both classification and diagnosis. Superficial incisional SSIs remain confined to the skin and the subcutaneous layer beneath. They usually surface within thirty days after an operation, showing up as localized redness, heat, swelling and at times a pus-laden discharge. When caught early, they tend to respond to oral antibiotics coupled with simple wound care. If they are not managed properly, they can slip deeper into the tissue. In contrast to infections, deep incisional SSIs invade the lower layers of soft tissue extending through the fascia into the muscle. They may not surface until as late as ninety days after the operation and when prosthetic material is present, the latency can stretch to a full year. Managing these infections almost always requires a return to the operating room for thorough debridement and in some cases the implanted hardware must be removed. Such infections carry a higher risk of morbidity and impose a heavy burden on the healthcare system. Organ/space surgical site infections are those that take root inside the body cavities or tissue planes a surgeon opened or handled. In operations, they can appear as an epidural abscess or discitis. In chest surgeries, the infection may spill into the space or the mediastinum. These infections are incredibly difficult, usually demanding weeks of intravenous antibiotics and often a surgical drainage procedure. Of the three SSI categories, organ/space infections carry the risk of life-threatening complications and death. When an SSI is left untreated—or when the initial therapy does not quite hit the mark—the infection can cascade into sometimes irreversible complications. A frequent fallout is dehiscence, the unsettling partial or complete separation of wound layers, which crops up in roughly 15 to 25% of infected spinal wounds. In cases involving instrumentation, hardware exposure becomes a serious concern, appearing in about 8 to 12% of deep-SSI instances. Moreover, insufficient early management can give rise to osteomyelitis—a bone infection—in approximately 10 to 15% of patients. Neurological complications—like abscesses or spinal-cord compression—appear in roughly 5% to 8% of spinal surgical-site infections and can leave patients with lasting deficits. In extreme cases, the infection may advance to sepsis, a systemic inflammatory response that occurs in about 3% to 5% of the more complicated infections and is linked to a high mortality rate. One of the notable clinical repercussions of surgical site infections is the need to return to the operating theater. A host of investigations has repeatedly demonstrated that infection dramatically raises the odds of an operation. Across the board, the re-operation rate after an SSI falls somewhere between 15% and 40%, depending on the infection’s depth and severity. When the infection is deep, the drive for revision becomes even more stark with re-operation rates soaring to roughly 60–80%. A quarter to a third of patients who receive spinal instrumentation end up needing the hardware removed. When infections prove stubborn or recur, another 20–30% of those patients often require more than one additional operation [142,143,144,145,146,147]. A year later, those same individuals showed markedly poorer functional outcomes than peers who escaped infection, underscoring the lingering toll SSIs can exact on quality of life and the overall recovery journey.

### 7.2. Healthcare Resource Utilization

SSIs place a load on healthcare systems chiefly by prolonging hospital stays and driving up resource use. Patients who develop an SSI typically remain admitted for two to three times longer than those who avoid complications. For infections, the extra time in the hospital usually falls in the eight-to-fourteen-day range, while deep SSIs can stretch the stay by roughly twelve to twenty-five days. A protracted hospitalization often emerges as a necessity when tighter monitoring, heightened nursing oversight, and a series of re-evaluations become indispensable. Patients fighting infections regularly undergo diagnostic workups—sophisticated imaging studies and a cascade of serial lab tests—to steer treatment decisions and keep a vigilant eye on how the therapy is responding. Treating SSIs is a demanding task, especially when the infection reaches deep structures or involves implanted devices. In 40% to 60% of deep SSI cases, surgeons must perform wound debridement to excise necrotic tissue and rein in the infection [130,138,140,146,148,149]. For patients who have received instrumentation, about a quarter to a third—approximately 25% to 35%—will need the hardware removed or revised. When the infection becomes particularly severe, complex wound-reconstruction procedures are often required, typically bringing plastic or reconstructive surgery teams into the fold. After leaving the hospital, ongoing wound management usually remains essential. This includes outpatient wound care and dressing changes which can stretch over weeks—or even months—depending on the wound’s complexity and the patient’s overall health. Antibiotics continue to be the backbone of SSI management, with regimens fine-tuned to the infection’s severity and depth. When an infection is only superficial, a course of antibiotics often suffices [119,146,148,149,150]. Deeper SSIs on the hand usually demand intravenous antibiotics typically administered over two to six weeks. A sizable proportion of patients enroll in outpatient antibiotic therapy (OPAT) programs, which let them receive IV treatment at home or in specialized care settings. From the moment the first dose is taken until the last, the patient’s condition must be watched through frequent clinical reviews paired with lab assessments—to confirm the therapy’s effectiveness and to catch any untoward effects or complications that may arise.

### 7.3. Functional Outcomes and Long-Term Consequences

SSIs may substantially degrade long-term performance and through a network of interwoven mechanisms give rise to lasting disability. A key challenge lies in delayed mobilization. Patients with SSIs often endure periods of bed rest and limited physical activity, which quickly lead to muscle deconditioning and a marked loss of strength. These constraints do not impede rehabilitation but also raise the risk of venous thromboembolism. Moreover, postponing mobilization typically means absences from work or school and a slower return to everyday life. Chronic pain frequently emerges as a disabling sequel. Ongoing ache at the wound site, heightened sensitivity, and the onset of pain—especially when nerves are directly involved—can dramatically erode quality of life. In individuals with infections, persistent low-back pain is a routine complaint, and the intended goals of surgery are often only partially achieved because of these complications. Surgical site infections can also give rise to complications, especially when they advance into epidural abscesses [119,151,152,153,154]. Such an abscess may compress the spinal cord, sparking nerve-root irritation, radiculopathy or—at the extreme—permanent neurological loss. These outcomes do not jeopardize the success of the original operation, but also leave patients with lasting functional impairments. The literature makes it clear that quality-of-life suffers noticeably when surgical-site infections occur. A slew of studies consistently report that SSIs depress outcomes across a range of domains. For example, individuals with an infection tend to score 15–25 points on the physical component of the SF-36 questionnaire. In parallel, their Oswestry Disability Index values are usually 20% to 30% higher, underscoring a greater degree of disability. On the EQ-5D utility, scores dip by 0.15–0.25 points, a shift that lays bare a marked slump in how patients perceive their own well-being. Infected individuals are two to three times more likely to report amplified depression and anxiety, underscoring the hefty psychological cost of these complications [116,118,151,152,155,156]. Long-term follow-up studies that extend two to five years after an SSI show that the impact stretches beyond the immediate postoperative period. Roughly a quarter to two-fifths of patients still grapple with pain or some level of disability. About one-in-six to one-in-five eventually need another operation. More striking, three-in-ten to half of those affected say they never regain the functional level they enjoyed before surgery. On top of that, two-in-ten to three-in-ten report persistent significant psychological distress as a lasting fallout of the infection. These results point to the layered consequences of SSIs—not merely the physical healing, but also the ripple effects on emotional health, long-term independence, and overall quality of life.

### 7.4. Risk Stratification and Predictive Modeling

Several validated tools have been developed to assess the risk of SSIs and to help guide prevention strategies based on individual patient and procedural characteristics. One widely used model is the National Nosocomial Infections Surveillance (NNIS) Risk Index. This tool combines three key variables: the ASA (American Society of Anesthesiologists) score, the classification of the surgical wound, and the duration of the operation [157]. Each factor is assigned a score, resulting in a total index ranging from 0 to 3. The associated infection rates increase with the cumulative risk score. For example, patients with a score of 0 have an approximate SSI rate of 1.5%, while those with a score of 1 have a 2.9% rate. The risk rises substantially with higher scores, reaching 6.8% at 2 points and 13.0% at 3 points. Another robust and commonly used tool is the ACS NSQIP (American College of Surgeons National Surgical Quality Improvement Program) Risk Calculator. Unlike the NNIS Index, this model generates procedure-specific risk estimates by incorporating a broad range of variables—up to 21 patient and surgical factors. It provides personalized risk percentages for a variety of postoperative outcomes, including SSIs. One of the key strengths of the NSQIP calculator is that it is regularly updated using data from a large national outcome database, ensuring that the predictions remain current and evidence-based. Together, these tools offer clinicians valuable insights that can inform patient counseling, guide the implementation of targeted prophylactic strategies, and support quality improvement efforts aimed at reducing the burden of surgical site infections.

Dedicated risk-assessment models fine-tuned for procedures now offer more precise predictions of SSI risk. The SpineSage Risk Model—one instrument—marshals a blend of patient demographics existing comorbidities and the idiosyncrasies of each surgical case. It churns out real-time calibrated risk numbers, furnishing clinicians with the evidence needed to judge if enhanced prophylaxis is warranted. Repeated validation across a variety of institutions has bolstered its credibility, earning it a place as a resource, in both academic investigations and bedside care. Another notable tool is the Surgical Site Infection Risk Index (SSIRI), a scoring system crafted for patients undergoing instrumented spinal fusion. It takes into account a handful of variables—body-mass index (BMI) diabetes status, the breadth of the fusion (particularly when multiple levels are involved), and the length of the operation. By weighing these factors, patients are placed into moderate or high-risk categories, which then steer how intensively perioperative infection-prevention measures are applied. When combined, the models sharpen the accuracy of risk forecasts for surgery and enable customized, data-backed strategies to prevent surgical site infections.

Making predictive risk models work in real-world settings hinges on a few essential pieces. First, they have to be woven into the electronic health record so clinicians can tap into them instantly. The model must provide a risk score on the spot and fire alerts to the care team. Once a patient is tagged as high-risk, a standardized protocol should spring into action, steering measures. Because treatment practices and patient populations are always evolving, the model needs regular validation and periodic updates to keep its predictions accurate. Beyond that, ensuring clinicians are well-trained and actively engaged is essential for the application and interpretation of the model’s outputs. When predictive modeling is linked directly to real-world outcomes, these tools provide a solid foundation for more systematic patient-specific strategies to prevent surgical site infections. This is especially valuable in high-risk populations, where early targeted interventions can markedly improve results.

## 8. Recommendations and Preventive Strategies

Having sifted through the research and applied a rigorous evidence-based lens, these recommendations lay out a practical framework for cutting down surgical site infections in spinal and thoracic operations. Arranged by the strength of the supporting data and their real-world relevance, the guidance offers clinicians actionable steps to improve patient outcomes [148,149,151].

### 8.1. Perioperative Antibiotic Management

#### 8.1.1. Level I Recommendations (Strong Evidence, High Confidence)

Intravenous antibiotics should be administered within 60 min prior to incision for most agents. For vancomycin or fluoroquinolones, this window can be extended to 120 min due to their prolonged infusion times [55,56,57,58,59,154]. Institutional protocols should be implemented, ideally with automated reminders, to ensure compliance with these timing guidelines. In high-risk procedures, administration within 30 min before incision should be considered to achieve optimal tissue concentrations [55,56,57,58,59,154].

Cefazolin (1–2 g IV) should be used as the first-line prophylactic antibiotic in patients without β-lactam allergies. For patients weighing more than 80 kg, the cefazolin dose should be increased to 2 g, and for those over 120 kg, a 3 g dose is recommended. Vancomycin should be reserved for patients who have documented MRSA colonization, severe β-lactam allergies, or are treated in institutions where MRSA prevalence exceeds 10%. Dual prophylaxis with cefazolin and vancomycin may be considered in high-risk patients who present with multiple infection risk factors [53,55,56,57,58,59,87,154].

Antibiotics should be re-dosed every 3 to 4 h during prolonged procedures, with cefazolin re-dosed every 4 h and vancomycin every 12 h. Re-dosing should also occur immediately if blood loss exceeds 1500 mL or if there are significant fluid shifts during surgery [46,50,51,52,54,100]. It is essential to maintain therapeutic antibiotic levels throughout the entire procedure, especially for surgeries lasting longer than 4 to 6 h.

Prophylactic antibiotics should be discontinued within 24 h after surgery unless there is a suspected active infection [31,38,39,49,50,51,52,133,154,158,159,160]. Extended prophylaxis beyond 24 h should be avoided in order to minimize the risk of Clostridioides difficile infection and the development of antimicrobial resistance. To ensure timely discontinuation, institutions should implement automatic stop orders and pharmacist-driven protocols.

#### 8.1.2. Level II Recommendations (Moderate Evidence, Reasonable Confidence)

Delivering Antibiotics Locally: In procedures involving spinal instrumentation placing 1–2 g of vancomycin powder directly into the wound is the norm [24,28,57,77,90,116,137,139] and can potentially provide additional protection in high-risk scenarios, but not all studies confirm the effectiveness of this method [85]. As the incision approaches closure, the surgical team might opt for an antiseptic irrigation—perhaps 0.35% povidone-iodine or 0.05% chlorhexidine—to keep microbes at bay [32,88]. Still, high-strength antiseptics should be shunned, given their potential to hamper the healing process. Populations with Specialized Characteristics: Given a prevalence of MRSA—whether among patients identified as high-risk or across a healthcare setting—screening and decolonization protocols ought to be instituted [20,21,49]. In immunocompromised individuals or anyone with documented colonization by organisms, extended-spectrum prophylaxis should be weighed as a means to bolster infection-prevention efforts [42,45,49,85,139].

### 8.2. Operative Technique and Risk Mitigation

#### 8.2.1. Level I Recommendations

Getting Surgery Lengths Right: Institutions should craft protocols aimed at trimming time, zeroing in on a handful of key tactics. These include preoperative planning and case-by-case preparation, smooth coordination and clear communication among all members of the surgical team, and the consistent use of standardized surgical approaches and techniques. For complex or extensive operations, staging the procedure can help keep each individual case shorter. Whenever feasible, the goal should be to finish the surgery in under four hours. If a longer operative time appears inevitable, enhanced prophylactic measures should be employed [13,17,21,22,23,39,161]. Reining in Blood Loss: A comprehensive approach to conserving blood during surgery needs to be put in place. It starts with getting patients’ hemoglobin up to levels before the procedure and giving tranexamic acid intra-operatively—a loading dose of about 15–20 mg/kg followed by a maintenance infusion of roughly 1–2 mg/kg per hour [21,22,23,39,54]. Throughout the case, meticulous attention to hemostasis and gentle tissue handling is essential. When appropriate, topical hemostatic agents can be administered. Provided it is safe and practical, controlled hypotension techniques may also be employed. Whenever practicable, aim to keep blood loss under 500 mL. If a larger volume is anticipated, heighten surveillance and apply appropriate prophylactic interventions [31,59,154].

#### 8.2.2. Level II Recommendations

Approaches that are minimally invasive: When the circumstances allow, opting for invasive techniques usually means less tissue injury, a shorter stint on the operating table and lower blood loss. Still, the benefit of incisions must be balanced against the risk of a longer procedure, especially when the case is intricate. Before any minimally invasive method is rolled out, it is vital that the surgical team has the requisite training and hands-on experience.

Key Considerations for Instrumentation: In particularly high-risk situations, the use of implants should be weighed as a way to curb infection risk. When the clinical picture permits, it is wise to trim the implant load as much as feasible. For surgeries that demand instrumentation, breaking the operation into separate stages can prove to be a useful strategy.

### 8.3. Advanced Wound-Management Technologies

#### 8.3.1. Level I Recommendations for NPWT

Indications of High Risk: In individuals harboring risk determinants, clinicians often recommend prophylactic NPWT. Notably, a body mass index (BMI) of 35 kg/m^2^ or higher [125,130,162], and a diagnosis of diabetes mellitus—especially when glycated hemoglobin (HbA1c) exceeds 7% [54,125,131]—signal the need for this intervention. Patients slated for spinal instrumentation that stretches across three or more levels [103,108] or whose procedures are expected to last more than four hours or result in blood loss exceeding 500 mL [4,29,54,94,95] are also considered high-risk.

Additional indications include revision surgery, prior radiation therapy and immunocompromised status. The technical implementation: Immediately after the primary wound closure is wrapped up in the operating room, NPWT should be set up without hesitation. The customary mode is a suction of around −125 mmHg. The dressing is left on for five to seven days though, the exact window is calibrated to the wound’s healing trajectory and the type of postoperative drainage observed [51,88,89,90,91]. Additionally, it is paramount that patients are furnished with NPWT instruction and that the follow-up regimen is unmistakably laid out.

#### 8.3.2. Level II Recommendations

##### Combination Therapies

In very high-risk patients, clinicians should consider combining NPWT with the application of intrawound antibiotics to enhance infection prevention [29,94,95]. The clinical effectiveness of this combined approach should be evaluated by analyzing institutional SSI rates alongside individual patient risk profiles.

##### Emerging Technologies

Ongoing monitoring of advancements in next-generation NPWT systems is important, particularly as newer models offer enhanced features that may improve outcomes. In selected cases, the use of antimicrobial-impregnated NPWT interfaces should also be considered to further reduce infection risk.

### 8.4. Patient Optimization and Risk Stratification

#### 8.4.1. Level I Recommendations

Fine-tuning the patient’s condition before the operation: Diabetic patients need their glucose control tightened so that HbA1c stays under 7% [3,10,44,125,126,127]. Smokers should be placed in cessation programs and remain smoke-free for at least four weeks before any operation. Nutritional deficits must be corrected, especially when serum albumin drops below 3.5 g/dL [1,5,7,8,15,61,162]. Moreover, chronic medical conditions should be optimally managed to enhance outcomes.

Assessing the Risks: When validated risk-stratification instruments—such as the National Nosocomial Infections Surveillance (NNIS) system, the American College of Surgeons National Surgical Quality Improvement Program (ACS NSQIP) or spine-specific models—are put into practice, they provide a gauge of a patient’s risk. The resulting scores should then act as a compass guiding the selection of prophylaxis protocols. It is essential that the risk assessment and its corresponding preventive strategy be recorded unambiguously in the patient’s record.

#### 8.4.2. Level II Recommendations

Weight Management: Whenever a postponement of surgery is clinically acceptable, encouraging weight loss in obese individuals is advisable. For patients slated for operations whose BMI exceeds 50 kg/m^2^, a bariatric surgery consultation merits consideration.

Immunomodulation: Immunosuppressive medication regimens should be meticulously refined in partnership with the specialists. When the clinical picture permits and safety is assured, brief temporary adjustments to the therapy can be entertained to lower infection risk and to improve outcomes.

### 8.5. Institutional Protocols and Quality Improvement

#### 8.5.1. Level I Recommendations

##### Bundled Care Protocols

Comprehensive SSI prevention bundles should be implemented as part of standardized perioperative care. These bundles should include preoperative skin decontamination using chlorhexidine gluconate, as well as the use of appropriate hair-removal techniques such as clipping instead of shaving. Maintaining perioperative normothermia, defined as a body temperature above 36 °C, is also essential. Glycemic control should be maintained throughout the perioperative period, with blood glucose levels kept below 180 mg/dL. Standardized antibiotic prophylaxis protocols must be followed consistently, and wound-management strategies should be adjusted based on the patient’s individual risk profile.

##### Quality Assurance

Institutions should establish regular audit and feedback mechanisms to monitor compliance with SSI prevention protocols [49,50]. A multidisciplinary case review process should be implemented for all SSI cases to identify areas for improvement. Surgical site infection rates should be tracked and benchmarked against national standards. Additionally, regular education and training updates should be provided to all members of the surgical team to reinforce best practices and maintain high-quality care.

#### 8.5.2. Level II Recommendations

##### Integrating Technology

Introducing EHR alerts paired with decision-support modules can nudge compliance toward infection-prevention gold standards. By tapping automated engines to orchestrate timing and redosing, one can secure a steady accurate rhythm throughout the perioperative timeline. In parallel, forging predictive-analytics tools would empower real-time risk appraisal and tailor interventions to the patient’s profile.

##### Innovation

Healthcare institutions should be partners in multi-institution quality-improvement initiatives that aim to cut down surgical site infections. Backing research into novel prevention approaches and emerging technologies—those that could meaningfully improve patient outcomes—remains crucial [58,59,115,116,117,118,125,129,130,154,155,163]. Clinical protocols meanwhile need to be revisited and refreshed regularly to stay aligned, with the evidence and best-practice standards.

### 8.6. Special Populations and Considerations

#### 8.6.1. Surgical Cases Marked by High Risk

During revision procedures, a bolstered prophylactic approach is recommended— a dual-antibiotic regimen coupled with prophylactic negative-pressure wound therapy (NPWT) [48,121,122,123,124]. For tumor resections, the antibiotic prophylaxis should be prolonged and wound surveillance heightened so that complications can be identified promptly. In deformity-correction surgeries, staged operations and intensive blood-conservation techniques should be contemplated when clinically appropriate [14,48,54,110,121,122,123,124].

#### 8.6.2. Pediatric Factors to Consider

Medication doses must be fine-tuned to a child’s weight class to keep the therapy both safe and effective. It is also crucial to give families in-depth education and steady support so they can manage postoperative care properly. When the case warrants it, we should employ NPWT protocols that reflect the unique physiological needs of pediatric patients.

#### 8.6.3. Resource-Limited Settings

In environments where resources are tight, the priority should be on high-impact actions—getting the timing of antibiotics right, selecting the most appropriate agent, and adhering to the correct treatment duration. Protocols need to be fashioned to match the resources and infrastructure on the ground. When advanced technologies are not available, the focus shifts to simple yet proven infection-prevention principles.

Grounded in evidence, these recommendations lay out an all-encompassing roadmap for staving off incision-site infections in spine and chest surgeries. Simultaneously, they leave plenty of wiggle-room for each institution to tweak them according to its resources, patient demographics, and precise quality-improvement objectives.

## 9. Future Directions and Emerging Technologies

The field of surgical site infection prevention is shifting fast thanks to technology, a clearer grasp of infection biology, and a push toward personalized care. Here, we outline the trends and forward-looking strategies that might overhaul SSI prevention in spinal and thoracic surgery; it can be associated with advanced biomaterials and antimicrobial technologies, artificial intelligence and predictive analytics, novel wound-management technologies, precision medicine approaches, and minimally invasive with robotic surgery techniques. Moreover, the integration of artificial intelligence into robotic surgical systems adds a new layer of sophistication to operative care. AI-powered tools can assist in the automated identification of critical anatomical structures, helping to avoid inadvertent injury and ensuring safer dissection. Real-time feedback during surgery allows for continuous adjustments to technique, promoting optimal outcomes. In addition, predictive modeling can support surgeons in planning the most effective surgical approach for each patient based on individual anatomy and procedural complexity. Together, these innovations point toward a future where technology plays a central role in improving surgical safety, precision, and infection prevention.

There are several critical research questions that remain unanswered and must be explored further to advance the field of SSI prevention. One of the most pressing areas involves determining the optimal protocols for NPWT, including ideal pressure settings, the recommended duration of use, and the most appropriate criteria for patient selection. Additionally, more systematic evaluations are needed to assess the potential synergistic effects of combining multiple prevention strategies, rather than evaluating each in isolation. Another important area of inquiry is the long-term impact of these preventive measures—not only in terms of infection rates but also in how they influence functional outcomes and quality of life over time. Equally important is the field of implementation science, which seeks to understand the real-world challenges to adopting evidence-based practices and to identify both the barriers and enablers that influence their successful integration into clinical workflows.

## 10. Limitations and Considerations

This review flags notable caveats that ought to be kept in mind when weighing its conclusions. The studies pulled together are anything but uniform— they span a range of designs which involve disparate patient cohorts and employ differing definitions and measurement approaches for outcomes. That patchwork of methods inevitably introduces variability, which can cloud the consistency and the side-by-side comparability of the reported results. Second, we cannot rule out the specter of publication bias—studies that report positive or significant outcomes are more likely to get published than those that yield negative or inconclusive findings. This tilt can warp the picture presented in the literature. Third, a lot of the studies we examined did not include any extended, long-term follow-up, making it hard to tell whether the interventions truly endure or have a lasting impact. Finally, differences in how institutions operate the clinical pathways they follow and the resources they have at their disposal can limit the generalizability of the results. What works in one setting does not automatically translate to another, especially when patient demographics or the underlying infrastructure differ.

## 11. Conclusions

Surgical site infections following spinal and thoracic procedures represent a complex clinical challenge that requires a comprehensive, evidence-based approach to prevention and management. This systematic review synthesized evidence from over 50 contemporary studies encompassing more than 25,000 patients to provide actionable insights for clinical practice.

The evidence shows that SSI risk is significantly influenced by modifiable intraoperative parameters, particularly surgical duration, blood loss, and extent of tissue dissection. These factors interact to create compound risk profiles that can be quantified using validated risk-assessment tools. Identification of high-risk patients enables targeted application of advanced prevention strategies, optimizing clinical outcomes.

### 11.1. Key Evidence-Based Conclusions

1. Antibiotic Prophylaxis Optimization: Proper timing (within 30–60 min), appropriate agent selection with weight-based dosing, and timely discontinuation (within 24 h) remain fundamental to SSI prevention. Intrawound antibiotics, particularly vancomycin powder in spinal instrumentation cases, can potentially provide additional protection in high-risk scenarios [3,10,20,21,22,23,25,34,39,40,56,58,62,85,101,154,163].
2. Operative Risk-Factor Management: Minimizing operative duration (<4 h when possible) and blood loss (<500 mL) significantly reduces SSI risk. These targets should guide surgical planning and technique refinement, with enhanced prophylaxis protocols for cases exceeding these thresholds [34,54,56,137,138,139,163].
3. Negative-Pressure Wound Therapy: Prophylactic NPWT is highly effective for high-risk patients, including those with BMI ≥ 35, diabetes mellitus, multilevel instrumentation, or anticipated prolonged procedures [48,118,119,120,121,122,123,124,125,126,127,128,130]. Evidence supports 5–7-day treatment protocols, with significant reductions in both superficial and deep SSI rates.
4. Risk Stratification: Use of validated risk-assessment tools enables personalized prevention strategies, targeting interventions to patients most likely to benefit [6,116,117,118,137,138,139,140,155]. This approach optimizes clinical outcomes while managing healthcare resources efficiently.
5. Bundled Care Approaches: Comprehensive prevention bundles that address multiple risk factors simultaneously demonstrate superior outcomes compared to isolated interventions [33,92,136,137,138,139,140,163]. Bundles should include preoperative optimization, standardized prophylaxis protocols, and risk-stratified wound-management strategies.

### 11.2. Final Recommendations

Adopting a risk-stratified multidisciplinary strategy is widely endorsed for curbing site infections (SSIs) in spinal and thoracic operations. It should begin with a rollout of evidence-based antibiotic prophylaxis, ensuring every patient receives the right preventive regimen both before the incision and during the postoperative phase. Then a systematic risk appraisal using validated tools lets the care team pinpoint those patients who sit at the higher-risk end of the SSI spectrum. For patients flagged as high-risk, advanced prophylactic options—such as NPWT or intrawound antibiotics—should be employed after a careful case-by-case assessment. Sustaining their benefit over the term calls for an active quality-improvement loop: systematic outcome tracking coupled with continual refinement of institutional protocols, as new evidence and performance insights emerge. Simultaneously, institutions should channel resources into both embracing and rigorously assessing technologies and frontier research, keeping the field moving forward. Integrating the scientific evidence creates a sturdy platform for effective SSI prevention strategies that can substantially boost patient outcomes. Looking ahead, a sustained focus on implementation science, emerging technologies and personalized medicine promises to sharpen our ability to ward off these postoperative complications. When these evidence-based tactics are applied reliably, surgeons can markedly cut the incidence of SSIs in spinal and thoracic procedures. The result is two-fold: patients enjoy outcomes while the healthcare system becomes more efficient and effective. Long-term success ultimately hinges on a commitment to both time-tested best practices and fresh innovation.

## Figures and Tables

**Table 1:** Intraoperative vancomycin powder dosing.

Patient Condition	Suggested Topical Vancomycin Dose
Normal renal function	1–2 g
Mild-to-moderate renal insufficiency	0.5–1 g
Severe renal insufficiency (GFR < 30)	≤0.5 g or avoid if IV vancomycin is used
Pediatric (<12 years)	Weight-based dosing not standardized; caution advised

Ref. [24] Luo H, Ren Y, Su Y, Xue F, Hong Z. Intraoperative vancomycin powder to reduce surgical site infections after posterior spine surgery: a systematic review and meta-analysis. EFORT Open Rev. 2022 Feb 15;7(2):109–121.

**Table 2:** Risk stratification framework for SSI prevention.

Risk Factor Category	Factor	Definition/Threshold
Patient Factors—Major	BMI ≥ 35 kg/m^2^	Severe obesity
	Uncontrolled diabetes	HbA1c ≥ 8.0%
	Active smoking	Within 4 weeks of surgery
	Immunosuppression	Steroids >10 mg/day or immunosuppressive drugs
	Previous SSI	History of surgical site infection
	Malnutrition	Albumin <3.5 g/dL or weight loss >10%
Patient Factors—Moderate	BMI 30–34.9 kg/m^2^	Moderate obesity
	Controlled diabetes	HbA1c 7.0–7.9%
	Age ≥ 65 years	Elderly patient
	Chronic kidney disease	eGFR < 60 mL/min/1.73 m^2^
	COPD	Chronic obstructive pulmonary disease
	Peripheral vascular disease	Documented PAD
Surgical Factors	Single-level spine	1 vertebral level
	2–4 level spine	2–4 vertebral levels
	>4 level spine/complex deformity	>4 levels or scoliosis correction
	Thoracic surgery (standard)	Lobectomy, segmentectomy
	Complex thoracic surgery	Bilobectomy, pneumonectomy
	Operative time >5 h	Extended procedure duration
	Revision surgery	Repeat operation
	Combined approach	Anterior–posterior spine surgery

Clinical decision algorithm for risk-stratified SSI prevention. The table guides clinicians through a systematic risk assessment process using validated scoring criteria, followed by evidence-based prevention strategies tailored to patient risk level.

**Table 3:** Proposed algorithm for immunosuppressed patients.

Risk Stratification	Preventive Strategy
Mild immunosuppression (e.g., low-dose steroids)	Standard systemic antibiotics + TVP
Moderate risk (e.g., methotrexate, biologics)	TVP + NPWT + extended antibiotics (48 h)
High risk (e.g., transplant patients, chemotherapy)	Full combination: TVP + NPWT + tailored extended antibiotic protocol (48–72 h)

## Data Availability

Data are contained within the article.
